# Circadian rhythm analysis using wearable-based accelerometry as a digital biomarker of aging and healthspan

**DOI:** 10.1038/s41746-024-01111-x

**Published:** 2024-06-04

**Authors:** Jinjoo Shim, Elgar Fleisch, Filipe Barata

**Affiliations:** 1 Centre for Digital Health Interventions, Department of Management, Technology, and Economics, ETH Zurich, Zurich, Switzerland; 2 Centre for Digital Health Interventions, Institute of Technology Management, University of St. Gallen, St. Gallen, Switzerland

**Keywords:** Prognostic markers, Biomarkers, Epidemiology, Prognostic markers

## Abstract

Recognizing the pivotal role of circadian rhythm in the human aging process and its scalability through wearables, we introduce CosinorAge, a digital biomarker of aging developed from wearable-derived circadian rhythmicity from 80,000 midlife and older adults in the UK and US. A one-year increase in CosinorAge corresponded to 8–12% higher all-cause and cause-specific mortality risks and 3–14% increased prospective incidences of age-related diseases. CosinorAge also captured a non-linear decline in resilience and physical functioning, evidenced by an 8–33% reduction in self-rated health and a 3–23% decline in health-related quality of life score, adjusting for covariates and multiple testing. The associations were robust in sensitivity analyses and external validation using an independent cohort from a disparate geographical region using a different wearable device. Moreover, we illustrated a heterogeneous impact of circadian parameters associated with biological aging, with young (<45 years) and fast agers experiencing a substantially delayed acrophase with a 25-minute difference in peak timing compared to slow agers, diminishing to a 7-minute difference in older adults (>65 years). We demonstrated a significant enhancement in the predictive performance when integrating circadian rhythmicity in the estimation of biological aging over physical activity. Our findings underscore CosinorAge’s potential as a scalable, economic, and digital solution for promoting healthy longevity, elucidating the critical and multifaceted circadian rhythmicity in aging processes. Consequently, our research contributes to advancing preventive measures in digital medicine.

## Introduction

The question “How old are you?” is a colloquial inquiry that generally pertains to an individual’s chronological age, representing the time elapsed since birth. Since the 1980s, however, researchers have recognized its imprecision as a surrogate measure for assessing the aging process^1^. Recent breakthroughs in aging research involve utilizing DNA methylation status to construct ‘epigenetic clocks’, which demonstrate accurate biological age estimation for various tissues and cell types throughout an individual’s lifespan^2–4^. This innovative approach links the aging process to our genetics and epigenetics, helping us understand how our bodies age and develop age-related morbidity and mortality, thus a more accurate indicator than chronological age^5^. Despite accumulating supportive evidence, for these clocks to reach their full potential, they must be easily accessible, affordable, and scalable as it ensures that the technology can be applied to a diverse range of populations, from individuals with specific medical conditions to those in good health interested in disease prevention. Regrettably, as of now, a method meeting these criteria has yet to emerge.

Circadian rhythm (CR), a self-perpetuating 24-hour oscillation, is gaining rapid recognition as a pivotal component of healthspan and biological aging^6–8^. CR actively governs essential biological and physiological functions such as sleep-wake cycles, body temperature, hormonal secretion, immune response, and cognitive and physical performance^9,10^. Disruptions to one’s normal rhythmicity are linked to increased susceptibility to infection and a higher likelihood of metabolic syndrome, diabetes, cardiovascular disease, cancer, depression, inflammatory diseases, and chronic inflammation^7,10–12^. Beyond its implications in diseases, the systemic effects of CR are intrinsically associated with the mechanisms of aging. CR emerges during early infancy and undergoes various changes through the lifespan and with aging^13^. With increasing age, the circadian system becomes fragmented and dampened in amplitude with a shift in phase, often leading to poor sleep quality and efficiency^14^. While many of these changes can be attributed to the impairment of normal rhythmicity that perturbs cellular functions and the release of hormones responsible for rhythmic activities, circadian disruption may occur secondarily to changes in the environment^15^. Studies consistently show that prolonged and repeated circadian disruption ultimately increases mortality risk and shortens longevity^16^. More recent work unveils that disrupted CR is additionally associated with decreased healthspan and advanced biological aging as estimated through blood biomarkers, underscoring the significant role of CR in healthy longevity as well as overall lifespan^17,18^.

Recent advancements in digital devices, such as wearables and smartwatches, offer scalable, unobtrusive, and long-term monitoring capabilities to assess and monitor an individual’s circadian rhythmicity^19^. Traditional CR assessment using the melatonin profile requires the continuous collection of blood or saliva samples for at least 24 h, which presents several challenges including high cost, labor-intensive and invasive sampling procedure, and infeasible protocol for large population studies^20^. Research-grade actigraphs, and most recently consumer-centric wearables, can characterize CR through continuous monitoring of an individual’s rest-activity circadian rhythms, sleep-wake cycles, and physical activity in a real-world setting^21–23^.

Considering the accumulating scientific evidence of circadian rhythm’s significant impact on health and aging, and the widespread accessibility of digital technology capable of monitoring it, we developed an approach that leverages 7-day wearable data to serve as a digital CR biomarker for the estimation of an individual’s biological aging and healthspan. Applying a rigorous evaluation scheme using two large independent cohorts, we comprehensively tested whether our CR biomarker (CosinorAge) reflects an individual’s biological aging process and predicts mortality, morbidity, and age-related functional decline. We elucidated underlying circadian characteristics contributing to advanced aging. Furthermore, we compared the predictive performance between circadian rhythmicity and physical activity in estimating biological aging. This research contributes to unlocking the digital potential to assess the aging process as it investigates the capability of accelerometry, a widely adopted technology in smartphones and smartwatches. Thus, it contributes to enabling population-wide primary prevention strategies and mitigating risks associated with advanced aging through digital devices.

## Results

### Population characteristics

Table 1 describes the baseline characteristics of participants from the UK Biobank (UKB) and the US National Health and Nutrition Examination Survey (NHANES). Of these 76,026 eligible participants in UKB (median age 58.0 years [IQR 51–63]), 45,911 (60.4%) were female and 30,115 (39.6%) were male. Participants were mostly white Caucasian origin (97.5%). 46,913 (61.7%) of participants had paid employment, and 5833 (7.7%) reported a shift work experience. The accelerometer-derived median sleep, sedentary, light intensity activity, and moderate-to-vigorous intensity activity (MVPA) durations were 8.3 h/day, 8.0 h/day, 4.3 h/day, and 0.5 h/day, respectively. The most common comorbid conditions were hypertension (23.4%), cancer (14.9%), and diabetes (3.5%). We applied a 70:30 split of the UKB data into the training cohort and internal validation cohort.

**Table 1:** Population characteristics

Median [IQR] or *n* (%)	Training (UKB; *n* = 53,218)	Internal Validation (UKB; *n* = 22,808)	External Validation (NHANES; *n* = 3371)
Age (years)	58.0 [51, 63]	58.0 [51, 63]	55.0 [47, 62]
Sex			
Female	32,151 (60.4)	13,760 (60.3)	1843 (54.7)
Male	21,067 (39.6)	9048 (39.7)	1528 (45.3)
Race/Ethnicity			
White	51,901 (97.5)	22,221 (97.4)	1194 (69.8)
Non-white	1317 (2.5)	587 (2.6)	2177 (30.2)
Townsend deprivation index	−2.50 [−3.84, −0.28]	−2.47 [−3.83, −0.23]	-
Baseline assessment center			
England	47,760 (89.7)	20,462 (89.7)	-
Wales	2015 (3.8)	864 (3.8)	-
Scotland	3443 (6.5)	1482 (6.5)	-
Education			
College or above	23,448 (44.1)	9899 (43.4)	1818 (63.6)
High school or equivalent	25,544 (48.0)	11,024 (48.3)	738 (21.0)
Less than high school	4226 (7.9)	1885 (8.3)	815 (15.4)
BMI			
Normal or underweight	21,694 (40.8)	9228 (40.5)	870 (24.6)
Overweight	21,850 (41.1)	9357 (41.0)	1113 (34.9)
Obese	9674 (18.2)	4223 (18.5)	1388 (40.6)
Smoking status			
Never	30,825 (57.9)	13,299 (58.3)	1818 (51.9)
Previous	18,867 (35.5)	8080 (35.4)	853 (27.3)
Current	3526 (6.6)	1429 (6.3)	700 (20.8)
Alcohol consumption			
Not current	2824 (5.3)	1228 (5.4)	1291 (29.5)
<3 times/week	24,248 (45.6)	10,272 (45.0)	1383 (45.6)
≥3 times/week	26,146 (49.1)	11,308 (49.6)	477 (19.8)
Unknown/Missing	0 (0.0)	0 (0.0)	220 (5.1)
Employment status			
Paid employment	32,809 (61.7)	14,104 (61.8)	1932 (64.4)
Unpaid	20,409 (38.3)	8704 (38.2)	1439 (35.6)
History of shift work			
No	49,107 (92.3)	21,086 (92.5)	-
Yes	4111 (7.7)	1722 (7.5)	-
Accelerometer-derived physical activity (hours/day)			
Sleep	8.29 [7.66, 8.97]	8.29 [7.67, 8.96]	7.00 [6.00, 8.00]
Sedentary behavior	7.95 [6.82, 9.03]	7.93 [6.81, 9.02]	11.53 [9.50, 13.23]
Light intensity activity	4.33 [3.43, 5.34]	4.33 [3.42, 5.32]	4.40 [3.23, 5.78]
MVPA	0.48 [0.23, 0.84]	0.49 [0.24, 0.84]	1.04 [0.56, 1.78]
Comorbidities			
Hypertension	12,430 (23.4)	5315 (23.3)	2002 (56.2)
Diabetes	1839 (3.5)	787 (3.5)	546 (12.6)
Cardiovascular diseases	175 (0.3)	64 (0.3)	340 (8.5)
Cancer	7936 (14.9)	3278 (14.4)	322 (12.2)
Respiratory disease	743 (1.4)	307 (1.4)	206 (6.6)
Neurodegenerative disease	103 (0.2)	40 (0.2)	-
Circadian rhythm characteristics			
MESOR (mg)	27.62 [23.02, 33.00]	27.56 [23.04, 33.06]	35.17 [28.51, 43.08]
Amplitude (mg)	23.53 [18.74, 29.43]	23.52 [18.70, 29.41]	13.67 [8.60, 19.66]
Acrophase (clock hour)	13:57 [13:15, 14:35]	13:54 [13:15, 14:35]	13:27 [12:04, 14:52]

% and IQR are adjusted for weights for NHANES.

*n* number of subjects, *IQR* inter-quartile range, *BMI* body mass index, *MVPA* moderate-to-vigorous intensity activity*, MESOR* midline estimating statistic of rhythm.

The NHANES served as an external validation cohort, which included 3371 eligible participants (median age 55.0 years [IQR 47–62]). 54.7% of participants were female, 69.8% were White race/ethnicity, and 64.4% had paid employment. Median sleep, sedentary, light intensity activity, and MVPA durations were 7.0 h/day, 11.5 h/day, 4.4 h/day, and 1.0 h/day, respectively. Common comorbidities comprised hypertension (56.2%), diabetes (12.6%), and cancer (12.2%).

In UKB, the median duration of follow-up was 8.1 years [IQR 7.5–8.6]. By the end of the follow-up, 2867 (3.8%) of 76,026 participants died (1999 from training and 868 from validation). In NHANES, 199 deaths (5.9%) were recorded, and a median follow-up was 6.9 years [IQR 5.9–7.9].

### Associations between CosinorAge and mortality risks

To evaluate the performance and utility of CosinorAge, we quantify the rate of aging estimated by our proposed approach using ‘CosinorAgeAdvance’. CosinorAgeAdvance is defined as the difference between CosinorAge and actual chronological age, following a similar naming convention employed in other biological age estimators. CosinorAgeAdvance value > 0 represents that participants’ estimated ages appear older than their actual chronological age, indicating a 5-year mortality risk greater than that predicted by chronological age, therefore indicating faster aging. Conversely, CosinorAgeAdvance value ≤ 0 indicates an equal or lower 5-year mortality risk and slower aging.

**Fig. 1:**
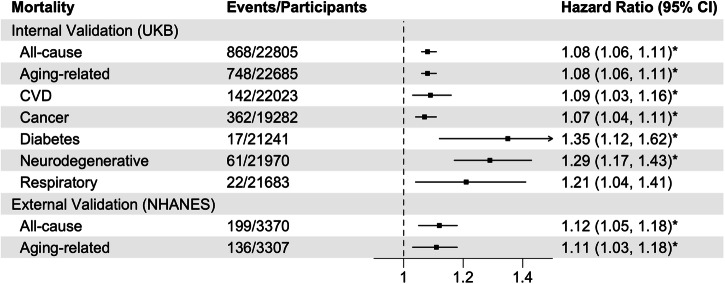
Associations between CosinorAge advancement and mortality risks. Cox proportional hazard regression models were adjusted for age, sex, ethnicity/race, Townsend deprivation index (UKB only), baseline assessment center (UKB only), education, employment, body mass index, smoking status, alcohol consumption, and comorbidities. Diabetes- and chronic respiratory disease-specific mortalities were adjusted for age to achieve model convergence. We account for complex survey design in NHANES. Participants with prevalent diseases corresponding to the related disease-specific mortalities were excluded from the relevant analyses. Aging-related mortality is defined as death resulting from any of the following diseases: CVD, cancer, diabetes, neurodegenerative, or chronic respiratory diseases. *p values remained significant (*p* < 0.007) after multiple tests with the Bonferroni correction.

First, we assessed the extent to which CosinorAge predicts the mortality risks in UKB and NHANES validation cohorts. Mortality stands as a key age-related outcome used in the evaluation of the biological age estimator. We observed that CosinorAgeAdvance was significantly associated with elevated risks of all-cause and aging-related cause-specific mortalities in multivariable Cox proportional hazards models adjusting for sociodemographic, lifestyle factors, and comorbidities. Specifically, a one-year increase in CosinorAgeAdvance was associated with an 8% increased risk of all-cause mortality (HR = 1.08, 95% confidence interval [CI] = 1.06–1.11) and an 8% increased risk of aging-related mortality (HR = 1.08, 95% CI = 1.06–1.11) in the UKB validation cohort (Fig. 1). When restricting to age-related disease-specific deaths, each one-year increase in CosinorAgeAdvance was associated with 9% (HR = 1.09, 95% CI = 1.03–1.16) and 7% (HR = 1.07, 95% CI = 1.04–1.11) elevated risks of cardiovascular disease (CVD) and cancer mortalities, respectively. Advanced CosinorAge was also significant with high neurodegenerative mortality risks (HR = 1.29, 95% CI = 1.17–1.43). In the NHANES validation cohort, CosinorAge was associated with slightly higher mortality risk as a one-year increase in CosinorAgeAdvance was associated with a 12% increased risk of all-cause mortality (HR = 1.12, 95% CI = 1.05–1.18) and 11% increased risk of aging-related mortality (HR = 1.11, 95% CI = 1.03–1.18). In stratified samples by sex, the associations between CosinorAge and mortality risks generally remain consistent as observed in the entire population. CVD- and diabetes-specific mortalities, however, appeared to be only significant among men (Supplementary Table 2).

### Associations between CosinorAge and disease incidences

**Fig. 2:**
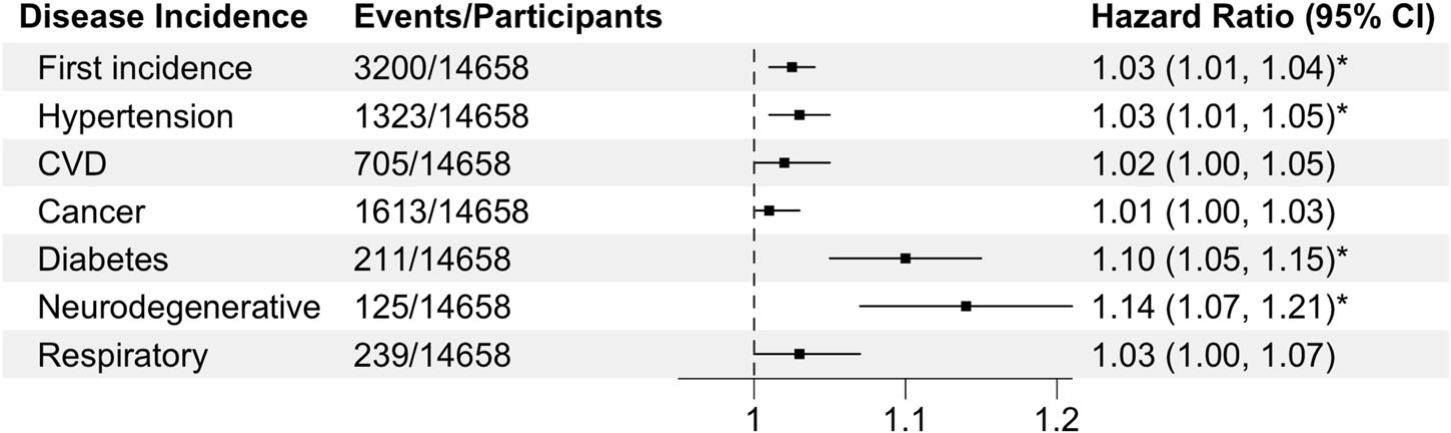
Associations between CosinorAge advancement and disease incidences. Cox proportional hazard regression models were adjusted for age, sex, ethnicity/race, Townsend deprivation index, baseline assessment center, education, employment, body mass index, smoking status, and alcohol consumption. Diabetes- and chronic respiratory disease-specific incidences were adjusted for age to achieve model convergence. Participants with prevalent diseases were excluded from the analyses. First incidence was defined as the first occurrence of any of the aforementioned diseases. *p values remained significant (*p* < 0.007) after multiple tests with the Bonferroni correction.

Next, we evaluated if CosinorAge predicts the future incidence of age-related conditions. Age-related morbidities serve as significant indicators for the health states of individuals, which were shown to be important in an individual’s trajectory of lifespan and healthspan. To this end, we included 14,658 participants without any diagnosis of such as hypertension, CVD, cancer, diabetes, neurodegenerative, and chronic respiratory diseases, during the eight years of follow-up from the UKB validation cohort. There was no relevant information available in NHANES. We found that each one-year increase in CosinorAgeAdvance was associated with 3% increased risk of first incidence of age-related disease (HR = 1.03, 95% CI = 1.01–1.04) and 3–14% elevated risks of specific diseases during the follow-up in the UKB validation cohort (Fig. 2). The highest incidence risk was associated with neurodegenerative disease (HR = 1.14, 95% CI = 1.07–1.21) followed by diabetes (HR = 1.10, 95% CI = 1.05–1.15) and hypertension (HR = 1.03, 95% CI = 1.01–1.05). Incident risks of CVD, cancer, and chronic respiratory diseases were not statistically significant after adjusting for covariates. In the sex-stratified analysis, the risks of first incidence were similar between men and women; however, future risks of diabetes, neurodegenerative disease, and chronic respiratory diseases appeared to be differential and were higher among women than men (Supplementary Table 3).

### Associations between CosinorAge and age-related functional performances

We further evaluated the effect of predicted age on multiple domains of functional performances such as self-rated health, handgrip strength, and health-related quality of life (HRQoL) measures. These metrics are indicatives of an individual’s perceived physical and mental health, serving as crucial predictors of morbidity, mortality, and increased hospitalization risk, directly influencing healthy longevity. Overall, CosinorAge was generally linked to unfavorable functional performance, indicated by poorer self-rated health, reduced handgrip strength, and declined HRQoL scores (Table 2). In the UKB validation cohort, participants with CosinorAgeAdvance values one-standard deviation (SD) older scored 0.09 units higher on the self-rated health score (poorer health), 0.01 units lower on the grip strength score, and 0.05 units lower on EQ-5D score (poorer HRQoL). In the NHANES validation cohort, we observed similar relationships as a one-SD increase in CosinorAgeAdvance was significantly associated with 0.10 units higher on the self-rated health score and 0.09 units higher on the ADL score (poorer HRQoL).

**Table 2:** Associations between CosinorAge advancement and age-related functional performances

Internal Validation (UKB)	Self-Rated Health		Grip Strength		EQ-5D	
	Coeff. (SE)	*p*-value	Coeff. (SE)	*p*-value	Coeff. (SE)	*p*-value
CAA (Continuous)	0.09 (0.006)	<0.001*	−0.01 (0.004)	0.001*	−0.05 (0.007)	<0.001*
CAA (Quartiles)						
Q1	Reference		Reference		Reference	
Q2	0.08 (0.016)	<0.001*	−0.02 (0.011)	0.145	−0.04 (0.021)	0.032*
Q3	0.12 (0.016)	<0.001*	−0.01 (0.013)	0.390	−0.09 (0.021)	<0.001*
Q4	0.26 (0.017)	<0.001*	−0.04 (0.012)	0.001*	−0.13 (0.021)	<0.001*

External Validation (NHANES)	Self-Rated Health		Grip Strength		ADL	
	Coeff. (SE)	*p*-value	Coeff. (SE)	*p*-value	Coeff. (SE)	*p*-value
CAA (Continuous)	0.10 (0.020)	<0.001*	0.01 (0.020)	0.477	0.09 (0.019)	<0.001*
CAA (Quartiles)						
Q1	Reference		Reference		Reference	
Q2	0.08 (0.070)	0.293	−0.02 (0.043)	0.650	0.03 (0.050)	0.494
Q3	0.09 (0.057)	0.125	0.07 (0.033)	0.044*	0.08 (0.047)	0.131
Q4	0.33 (0.067)	<0.001*	0.01 (0.053)	0.808	0.23 (0.053)	<0.001*

Generalized linear models were adjusted for age, sex, ethnicity/race, Townsend deprivation index (UKB only), baseline assessment center (UKB only), education, employment, body mass index, smoking status, alcohol consumption, and comorbidities. We account for complex survey design in NHANES.

*CAA* CosinorAgeAdvance, *SE* standard errors, *EQ-5D* European Quality of Life-5 Dimensions 5-levels*, ADL* Activities of Daily Living, *HRQoL* Health-related quality of life.

*p values < 0.05. We standardized the CAAs and outcomes to have a mean value of 0 and a standard deviation of 1.

**Fig. 3:**
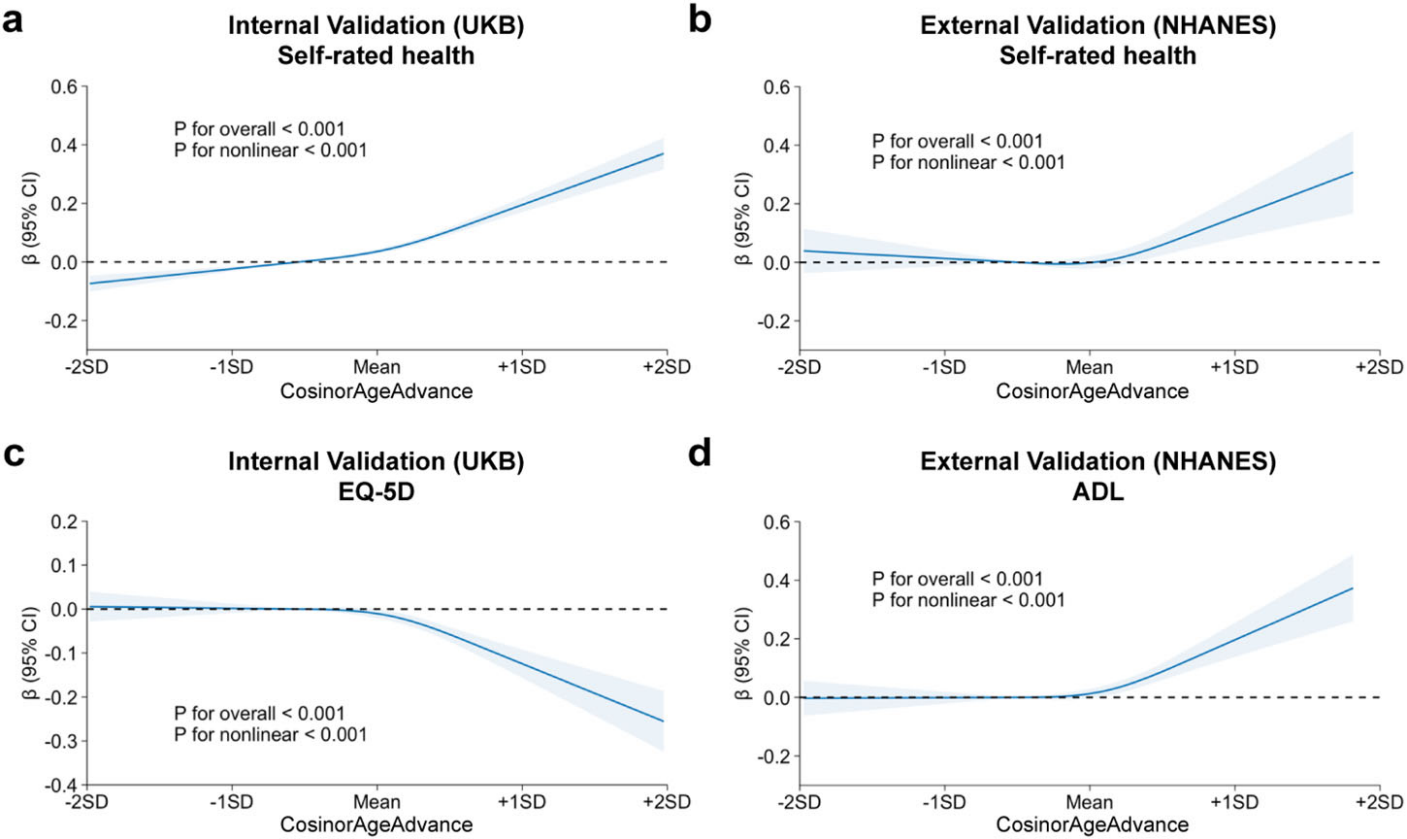
Dose-response relationships between CosinorAge advancement and age-related functional performances. **a**, **b** Self-rated health in the UKB and NHANES validation cohorts. **c**, **d** Health-related quality of life metrics in the UKB (EQ-5D) and NHANES (ADL) validation cohorts. Solid line: Point estimation; Shaded areas: 95% confidence limits. Restricted cubic spline regression models with knots at 25th, 50th, and 75th percentiles. Models were adjusted for age, sex, ethnicity/race, Townsend deprivation index (UKB only), baseline assessment center (UKB only), education, employment, body mass index, smoking status, alcohol consumption, and comorbidities.

Interestingly, we found that associations with self-rated health were non-linear across quartiles of CosinorAgeAdvance. For instance, the beta coefficients of self-rated health score in Q2, Q3, and Q4 quartiles (fast agers) compared to Q1 quartile (slow agers) were 0.08, 0.12, and 0.26 in the UKB validation cohort and 0.08, 0.09, and 0.33 in the NHANES validation cohort, showing a sharp increase in the beta coefficients among participants with an age advance above the mean (Fig. 3). Similarly, the beta coefficients of HRQoL measures in Q2, Q3, and Q4 were −0.04, −0.09, and −0.13 in the EQ-5D score (lower EQ-5D = poorer health) from the UKB validation cohort, and 0.03, 0.08, and 0.23 in the ADL score (higher ADL = poorer health) from the NHANES validation cohort.

We did not find a robust association between CosinorAge and grip strength, except among the top 25% of fast agers had significantly lower grip strength in the UKB validation cohort.

In the sex-stratified analyses, we observed variable impacts of advanced CosinorAge largely vary by functional outcomes (Supplementary Table 4). First, a decline in self-rated health was significantly associated with advanced CosinorAge in both sexes, exhibiting similar increasing trends across CosinorAgeAdvance quartiles. In the external validation cohort, we observed similar associations with statistical significance achieved only when comparing the top CosinorAgeAdvance quartile (fastest agers) to the reference group (slowest agers). Next, grip strength was significantly associated with advanced CosinorAge only among females in the internal validation cohort. Last, advanced CosinorAge was significantly associated with declined HRQoL, yet the gradient declining effect of EQ-5D score was observed among females but not in males in the internal validation cohort, indicating the observed associations are largely differential by sex and the rate of aging. In the external validation cohort, the ADL score was associated with advanced CosinorAge, and the top quartile (fastest agers) showed significantly poorer HRQoL measures compared to the reference group.

### Associations between CosinorAge and established biological age

Next, we asked whether our wearable-derived CosinorAge and other commonly used blood biomarker-based biological age estimators quantify the same aging process, thus assessing its complementary potential to the existing epigenetic clock.

**Fig. 4:**
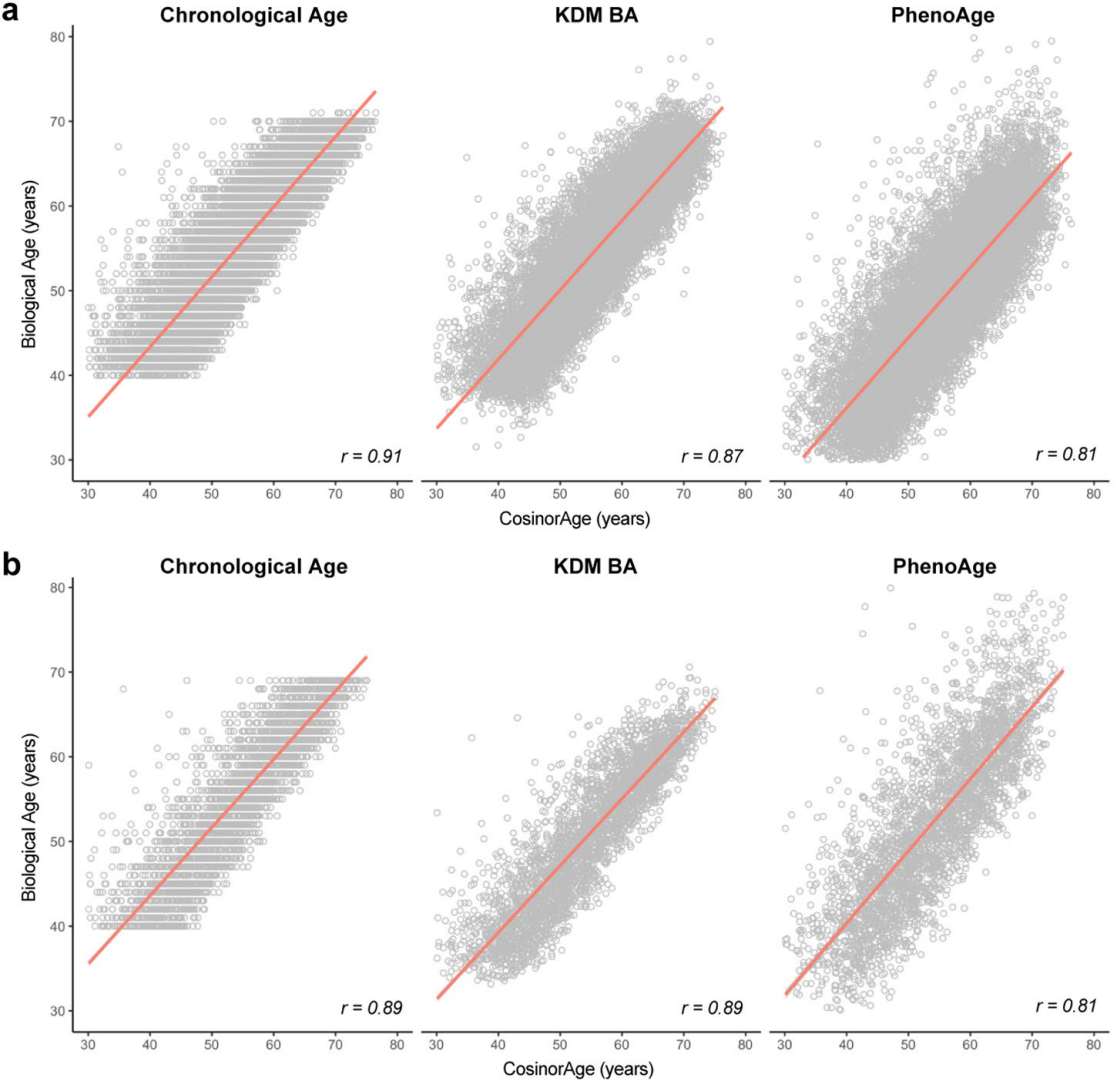
Associations between CosinorAge and established biological age measures. **a** Correlations with chronological age, Klemera-Doubal biological age (KDM BA), and phenotypic age (PhenoAge) in the UKB internal validation cohort. **b** Correlations with chronological age, KDM BA, and PhenoAge in the NHANES external validation cohort. *r* Pearson’s correlation coefficient.

We identified a subset of individuals with complete blood biomarker information, obtained Klemera-Doubal biological age (KDM BA) and phenotypic age (PhenoAge), and computed correlations between CosinorAge and each of the biological aging measures in UKB and NHANES validation cohorts (Fig. 4). CosinorAge was strongly correlated with both KDM BA and PhenoAge with *r* = 0.87 (KDM, *p* < 0.001) and *r* = 0.81 (PhenoAge, *p* < 0.001) in the internal validation set and *r* = 0.89 (KDM, *p* < 0.001) and *r* = 0.81 (PhenoAge, *p* < 0.001) in the external validation set. When stratified by sex, the correlations between CosinorAge and other biological age estimators remain consistently robust, with values ranging ranges from 0.80 to 0.90 (Supplementary Fig. 2). KDM BA exhibited a slightly higher correlation with CosinorAge compared to PhenoAge across diverse cohorts.

### Explainability of CosinorAge and underlying factors in its predictions

To elucidate the factors contributing to our age prediction and advanced aging, we examined detailed circadian characteristics of participants falling within the top 25% (fast agers) and bottom 25% (slow agers) of CosinorAgeAdvance, utilizing the internal validation cohort. To mitigate the impact of chronological age on changes in circadian rhythmicity, we categorized individuals based on the age at baseline into four groups: <45 years, 46–55 years, 56–65 years, and >65 years. We observed diverse changes in circadian parameters among various age groups when comparing different rates of aging based on CosinorAge (Table 3).

**Table 3:** Advanced CosinorAge and underlying circadian characteristics by age groups

	MESOR (mg)			Amplitude (mg)			Acrophase (clock hour)		
Age Group	Fast agers	Slow agers	Δ	Fast agers	Slow agers	Δ	Fast agers	Slow agers	Δ
<45 years	23.8	41.9	−18.1	17.1	37.4	−20.3	14:19	13:54	+25 min
46-55 years	22.1	39.4	−17.3	16.6	35.8	−19.2	14:10	13:54	+16 min
56-65 years	19.3	35.5	−16.2	15.7	33.8	−18.1	13:57	13:43	+14 min
>65 years	17.9	33.5	−15.6	14.4	31.6	−17.2	13:43	13:36	+7 min

For instance, in the elderly population (aged >65 years) undergoing fast aging, there was a substantial reduction in the median amplitude and MESOR levels compared to their peers of the same chronological age experiencing slower aging. The difference in the median acrophase, however, was less pronounced (13:43 vs. 13:36). Among the youngest age group (aged <45 years), fast agers not only exhibited a reduction in amplitude and MESOR values but also a significant shift in acrophase, with a delay of 25 min in the median peak activity time when comparing fast agers to slow agers (14:19 vs. 13:54). While shifts in peak timing were observed across all age groups, the disparities among CosinorAgeAdvance quartile groups were largest among young participants then decreased with increasing age. We also observed that young adults (aged <45 years) experiencing advanced aging displayed substantially lower amplitude and MESOR levels than old adults (aged >65 years) undergoing slow aging.

In the sex-stratified cohort, the observed associations remained consistent. Both sexes demonstrated a more substantial delay in peak time among young individuals experiencing fast aging, while a decline in amplitude and MESOR predominantly contribute to advanced aging in the elderly with advanced aging. Acrophase timing displayed variations with sex (Supplementary Table 5). In males aged 55 years or younger, fast agers’ peak timing occurred approximately a half hour later than that of slow agers, whereas the difference was less prominent in similarly aged females. Conversely, in participants aged older than 55 years, females exhibited a larger difference in acrophase timing compared to males.

### Predictive performance comparison between circadian rhythm-based CosinorAge and physical activity-based PA age

We conducted a comprehensive evaluation of the predictive capabilities of circadian rhythmicity-based age (CosinorAge) in comparison to physical activity in the internal validation set. Consistent with previous research^24–28^, we utilize moderate-to-vigorous physical activity (MVPA) as the metric to quantify physical activity. Subsequently, we develop an age metric, termed PA Age (physical activity-based age), employing the same methodology to develop CosinorAge, followed by a comparative analysis of both age predictors across various metrics.

CosinorAge exhibits a consistently higher accuracy in mortality risk prediction compared to PA Age (Table 4). The difference in C-index between CosinorAge and PA Age ranged from 0.004 to 0.116, indicating CosinorAge’s additional predictive power from 0.4% to 11.6% over PA Age. The most pronounced increment in predictive power was observed with neurodegenerative disease mortality (Δ C-index = 11.6%), followed by CVD mortality (Δ C-index = 5.9%), aging-related mortality (Δ C-index = 5.3%), all-cause mortality (Δ C-index = 5.2%), and cancer mortality (Δ C-index = 5.1%). In terms of AIC comparison, the CosinorAge model showed superior goodness of fit to the PA Age model, evidenced by substantial reductions in AIC values across all-cause mortality (Δ AIC = −80.5), aging-related mortality (Δ AIC = −67.8), cancer mortality (Δ AIC = −23.3), neurodegenerative disease mortality (Δ AIC = −21.8), and CVD mortality (Δ AIC = −15.8).

**Table 4:** Predictive performance of CosinorAge vs. PA Age for mortality and disease risks

Mortality	CosinorAge C-index (95% CI)	PA Age C-index (95% CI)	Δ C-index	CosinorAge AIC	PA Age AIC	Δ AIC
All-cause mortality	0.598 (0.591, 0.604)	0.546 (0.540, 0.552)	+0.052	17,117.5	17,198.0	−80.5
Aging-related	0.594 (0.588, 0.601)	0.541 (0.534, 0.548)	+0.053	14,743.7	14,811.5	−67.8
CVD	0.613 (0.600, 0.629)	0.554 (0.539, 0.572)	+0.059	2781.8	2797.6	−15.8
Cancer	0.573 (0.564, 0.583)	0.522 (0.512, 0.531)	+0.051	7044.3	7067.6	−23.3
Diabetes	0.735 (0.697, 0.775)	0.731 (0.498, 0.765)	+0.004	318.1	319.1	−1.0
Neurodegenerative	0.670 (0.647, 0.696)	0.554 (0.529, 0.581)	+0.116	1175.3	1197.1	−21.8
Respiratory	0.674 (0.533, 0.709)	0.660 (0.619, 0.701)	+0.014	424.6	423.2	+1.4
**Disease Incidence**	**CosinorAge C-index (95% CI)**	**PA Age C-index (95% CI)**	**Δ C-index**	**CosinorAge AIC**	**PA Age AIC**	**Δ AIC**
First incidence	0.522 (0.518, 0.526)	0.503 (0.500, 0.507)	+0.019	60,221.4	60,241.2	−19.8
Hypertension	0.534 (0.529, 0.540)	0.521 (0.516, 0.526)	+0.013	25,004.1	25,017.6	−13.5
CVD	0.530 (0.522, 0.538)	0.501 (0.493, 0.508)	+0.029	13,400.7	13,406.6	−5.9
Cancer	0.500 (0.495, 0.505)	0.523 (0.519, 0.529)	−0.023	30,565.3	30,561.1	+4.2
Diabetes	0.627 (0.614, 0.637)	0.590 (0.576, 0.603)	+0.037	3958.4	3979.8	−21.4
Neurodegenerative	0.619 (0.601, 0.638)	0.529 (0.511, 0.548)	+0.09	2347.2	2359.3	−12.1
Respiratory	0.528 (0.516, 0.541)	0.554 (0.542, 0.567)	−0.026	4541.5	4538.7	+2.8

The 95% CIs of C-index is computed using 1000 bootstrap samples.

*C-index* Concordance index, *AIC* Akaike information criteria.

**Fig. 5:**
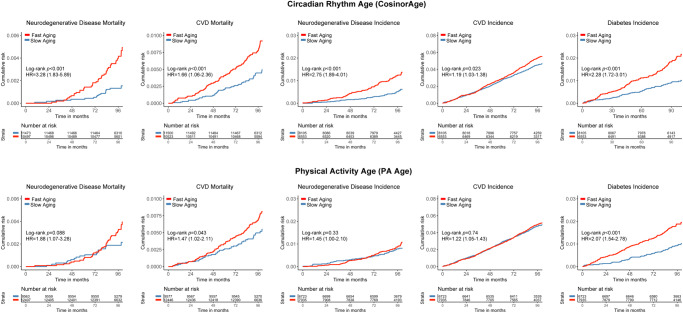
Cumulative risks of mortality and disease incidence of neurodegenerative disease, CVD, and diabetes stratified by the rate of aging. Fast aging is defined by CosinorAgeAdvance >0 or PA Age Advance >0. Slow aging is defined by CosinorAgeAdvance ≤0 or PA Age Advance ≤0. HR and 95% CI are obtained from Cox proportional hazards models adjusted for age, sex, ethnicity/race, Townsend deprivation index, baseline assessment center, education, employment, body mass index, smoking status, alcohol consumption, and comorbidities.

When considering the rates of aging, fast circadian rhythm aging exhibited more robust associations with mortality outcomes compared to physical activity aging, evidenced by the Kaplan-Meier plots and Cox regressions (Table 5 and Fig. 5). Specifically, fast circadian rhythm aging was statistically significantly associated with increased risks of CVD, diabetes, and neurodegenerative mortalities, whereas these associations are not significant with fast physical activity aging.

**Table 5:** Advanced CosinorAge vs. PA Age with mortality and disease risks

Mortality	CosinorAgeAdvance > 0 HR (95% CI)	PA Age Advance > 0 HR (95% CI)
All-cause mortality	1.55 (1.34, 1.78)*	1.35 (1.17, 1.56)*
Aging-related	1.50 (1.29, 1.74)*	1.35 (1.16, 1.58)*
CVD	1.66 (1.16, 2.36)*	1.47 (1.02, 2.11)
Cancer	1.32 (1.06, 1.63)	1.27 (1.02, 1.59)
Diabetes	5.46 (1.57, 19.02)*	2.59 (0.84, 7.94)
Neurodegenerative	3.28 (1.83, 5.89)*	1.88 (1.07, 3.28)
Respiratory	2.42 (0.98, 5.93)	4.88 (1.44, 16.48)
**Disease Incidence**	**CosinorAgeAdvance** > **0** **HR (95% CI)**	**PA Age Advance** > **0** **HR (95% CI)**
First incidence	1.15 (1.07, 1.24)*	1.11 (1.03, 1.19)*
Hypertension	1.12 (1.01, 1.26)	1.18 (1.05, 1.32)*
CVD	1.19 (1.03, 1.38)	1.22 (1.05, 1.43)
Cancer	1.04 (0.94, 1.15)	0.97 (0.88, 1.08)
Diabetes	2.28 (1.72, 3.01)*	2.07 (1.54, 2.78)*
Neurodegenerative	2.75 (1.89, 4.01)*	1.45 (1.00, 2.10)
Respiratory	1.31 (1.01, 1.69)	1.58 (1.21, 2.06)*

Cox proportional hazard regression models were adjusted for age, sex, ethnicity/race, Townsend deprivation index, baseline assessment center, education, employment, body mass index, smoking status, and alcohol consumption. Diabetes- and chronic respiratory disease-specific incidences were adjusted for age to achieve model convergence.

*p values remained significant (*p* < 0.007) after multiple tests with the Bonferroni correction.

CosinorAge also demonstrated superior predictive power for predicting disease incidence. The highest additional predictive power of CosinorAge was observed for predicting neurodegenerative disease (Δ C-index = 9%), followed by diabetes (Δ C-index = 3.7%), CVD (Δ C-index = 2.9%), and first incidence (Δ C-index = 1.9%). In terms of AIC comparison, CosinorAge model was a preferred model for predicting diabetes (Δ AIC = −21.4), first disease incidence (Δ AIC = −19.8), hypertension (Δ AIC = −13.5), neurodegenerative disease (Δ AIC = −12.1), and CVD incidence (Δ AIC = −5.9).

When evaluating the rate of aging with disease incidence by stratifying individuals into fast aging or slow aging groups based on their aging rate, both rapid circadian aging and rapid physical activity aging are significantly associated with elevated risks of initial incidence and diabetes risk. However, these associations are stronger in CosinorAge. Moreover, fast circadian rhythm aging is significantly linked to increased risks of neurodegenerative incidences, while it is not found significant in physical activity aging. Conversely, hypertension and respiratory disease incidences are only significantly associated with physical activity aging.

### Associations between CosinorAge and neurodegenerative disease subtypes

**Fig. 6:**
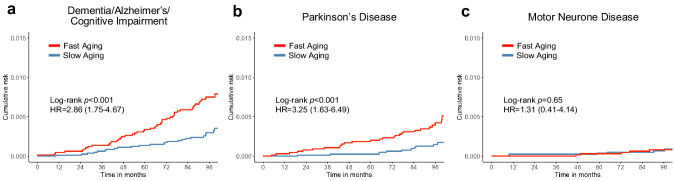
Cumulative risk of neurodegenerative disease subtypes stratified by the rate of aging. **a** Dementia/Alzheimer’s/Cognitive impairment. **b** Parkinson’s Disease. **c** Motor neurone disease. Fast aging is defined by CosinorAgeAdvance >0. Slow aging is defined by CosinorAgeAdvance ≤0. HR and 95% CI are obtained from Cox proportional hazards models adjusted for age, sex, ethnicity/race, Townsend deprivation index, baseline assessment center, education, employment, body mass index, smoking status, alcohol consumption, and comorbidities.

As shown with robust predictive performance, we further elucidated the effects of circadian rhythm aging on neurodegenerative disease risk. We divided the incident neurodegenerative cases into three sub-disease categories: (i) Dementia/Alzheimer’s/Cognitive impairment; (ii) Parkinson’s disease; and (iii) Motor neuron disease. Dementia, Alzheimer’s, and cognitive impairment are collectively grouped together due to a shared self-report question pertaining to these conditions. In Fig. 6, we demonstrated that individuals undergoing fast circadian rhythm aging have 2.86 times elevated risks of developing dementia/Alzheimer’s/cognitive impairment (HR = 2.86, 95% CI = 1.75–4.67) and 3.25 times elevated risks of developing Parkinson’s disease (HR = 3.25, 95% CI = 1.63–6.49). However, we did not observe a significant association between advanced CosinorAge with motor neurone disease incidence (HR = 1.31, 95% CI = 0.41–4.14).

We also examined whether individuals who eventually develop neurodegenerative diseases exhibit a higher prevalence of sleep disturbance at baseline before developing diseases. We found that the prevalence of sleep disturbance was notably higher among those who eventually develop neurodegenerative diseases compared to those who do not. Specifically, 31% of participants who eventually develop neurodegenerative diseases reported prevalent insomnia at baseline, contrasting with only 25% of those who remained free from such diseases. When considering insomnia, daytime dozing, and snoring collectively, we observed that 59% of participants who eventually develop neurodegenerative diseases exhibited sleep disturbance traits at baseline, in contrast to 49% of those who remain unaffected by neurodegenerative disease.

## Discussion

In this study, we introduced CosinorAge, a digital biomarker for aging and healthspan utilizing wearable-derived circadian rhythmicity in population-based cohorts of 80,000 midlife and older adults in the UK and the US. Our results demonstrated CosinorAge as a general marker of longevity and healthspan, as the advanced CosinorAge predicted the risks of prospective incidence of age-related diseases and mortality and identified functional vulnerabilities independent of demographics, socioeconomic covariates, and comorbidities with high accuracy. Having the identical chronological age, adults with one-year higher CosinorAge exhibited an 8–12% elevated risk of mortality and a 3–14% increased risk of age-related diseases compared to their peers who were estimated to be one-year biologically younger. Increased CosinorAge was also strongly associated with a significant decline in self-rated health (range −8 to −33%), grip strength (−1 to −4%), and HRQoL scores (−3 to −23%), indicating the loss of resilience and physical functioning. These relationships were non-linear and sexually dimorphic. Furthermore, we observed high correlations and the compatibility potential of CosinorAge with established Klemera-Doubal biological age and phenotypic age. We discovered notable age-specific variations in circadian peak timing associated with the different rates of biological aging. Circadian rhythm-based CosinorAge outperformed physical activity in predicting both mortality and disease incidence risks. Together, these findings help establish a new link connecting the critical and multifaceted role of circadian rhythmicity with aging processes, unveiling valuable insights for the general public as well as healthcare professionals.

Our study differed from prior studies and improved the current methodology with several contributions. Foremost, our results demonstrated CosinorAge as a general biomarker for longevity, as it not only predicts mortality, but serves as a highly informative metric in assessing age-related vulnerability, functional declines, and the prospective incidence of age-related diseases. We also newly reported the heterogeneous impact of circadian parameters associated with the biological aging process. Fast agers exhibited a similar rate of reduction in circadian amplitude and MESOR when compared to slow agers across all age groups; however, young (<45 years) and fast agers displayed a substantially delayed acrophase with a 25-minute difference in peak timing compared to slow agers, whereas the effect diminished to a 7-minute difference in older adults (>65 years). Our results revealed that the influence of circadian acrophase on biological aging was more pronounced in younger individuals, suggesting a plausible link between circadian misalignment or disruption and the adverse health implications associated with the aging process. This aligns with recent research, which has shown that young adults with circadian disturbance were seemingly healthy with no comorbid conditions but in fact, exhibited elevated inflammation and accelerated biological aging^18^.

Another distinctive contribution of our study lies in demonstrating a substantial improvement in predictive performance by integrating circadian rhythmicity in the estimation of biological aging over physical activity. To achieve this, we developed an age metric termed “PA Age” by utilizing MVPA information, employing a methodology analogous to that used to develop CosinorAge. The emergence of the PA Age is unprecedented, constituting a unique contribution to the existing literature. Our approach utilizes Gompertz modeling to ascertain PA Age derived from MVPA, thereby facilitating a fair comparative analysis between PA Age and the circadian rhythmicity-based age, CosinorAge. We demonstrated a noteworthy increase in predictive power with CosinorAge for mortality and incidence, particularly related to CVD, diabetes, and neurodegenerative disease mortalities and incidences. Fast circadian rhythm aging exhibits stronger associations with outcomes compared to fast physical activity aging. These findings align with the recent studies that emphasize the pivotal role of circadian timing in cardiovascular incidence, extending its relevance to both the general population and diabetic patients^29,30^. In these investigations, researchers have demonstrated that circadian abnormalities, characterized by reduced amplitude, delayed acrophase, low MESOR, and low rhythm stability, correlated with elevated risks of CVD, atrial fibrillation, as well as all-cause and CVD mortalities. Moreover, our prior research also demonstrates the importance of circadian rhythm on health outcomes. Despite engaging in high physical activity, the misalignment or circadian timing can lead to increased inflammation, advanced biological aging, and elevated mortality risk^18^, underscoring the critical role of circadian timing in health and longevity.

Neurodegenerative diseases, which demonstrate CosinorAge’s most significant predictive power for both mortality and disease incidence compared to PA Age, warrant further investigation. We found that fast circadian rhythm aging is significantly associated with a 2.86 and 3.25-fold increase in future dementia/Alzheimer’s/cognitive impairment and Parkinson’s disease, respectively. We also observed a substantially higher prevalence of sleep disturbance at baseline among individuals who later develop neurodegenerative diseases compared to those who do not. This suggests that sleep disturbances may be associated with undiagnosed neurodegenerative conditions and serve as early signs of neurodegenerative diseases. Circadian rhythm disturbances have been described in both preclinical and clinical neurodegenerative diseases, yet no biomarker incorporating circadian function to assess these conditions has emerged^31–33^. Our study presents new evidence, showing that biological age estimated using circadian rhythmicity derived from wearable accelerometry may hold potential for assessing future risks of neurodegenerative diseases.

CosinorAge marks a pioneering study to predict biological age based on digital circadian rhythmicity through wearables. A large body of evidence suggests the emerging link between the circadian clock, epigenetics, and age-related adverse outcomes; however, as of today, no biological clock has used passive, free-living sensing of physiological and behavioral signals from digital devices to assess circadian rhythm as a surrogate biomarker of aging and healthspan.

Recent advancements in digital devices, such as wearables and smartwatches, offer scalable, unobtrusive, and long-term monitoring capabilities to assess and monitor an individual’s circadian rhythmicity^19^. Traditional CR assessment using the melatonin profile requires the continuous collection of blood or saliva samples for at least 24 h, which presents several challenges including high cost, labor-intensive and invasive sampling procedure, and infeasible protocol for large population studies^20^. Wearable devices have emerged as viable alternative methods for characterizing CR through continuous monitoring of an individual’s rest-activity circadian rhythms, sleep-wake cycles, and physical activity in a real-world setting. In line with this, researchers have employed research-grade actigraphs, and most recently consumer-centric wearables, to investigate adverse effects regarding circadian rhythmicity and activity levels^21–23^. Personal digital wearables and smartwatches offer unparalleled potential for circadian rhythm assessment and age prediction with several advantages. These devices are scalable and easily accessible solutions enabling remote, continuous, and unobtrusive monitoring of activities and circadian cycle for an extended period, as opposed to invasive sample collection and costly laboratory analysis required in the current state-of-the-art biological age estimators. In addition, longitudinal monitoring capabilities provide a comprehensive trajectory of circadian health, supplying valuable information about changes in health state and personalized health optimization. Taken together, implementing CosinorAge for smartwatch data could be a scalable, economic, and digital solution to promote healthy longevity and circadian health. As the adoption of digital wearables continues to rise, their integration contributes significantly to advancing primary prevention efforts in the ever-growing aging population, presenting diverse clinical and public health implications.

To date, there is no gold standard for biological age estimator. CosinorAge algorithm based on proportional hazard models with Gompertz distribution brings several advantages. First, biological age estimators built with mortality are considered to be superior to chronological age predictor, and the Gompertz model is one of the most reliable parametric approaches for modeling human mortality and has been widely implemented in various populations. Second, the parameters in the Gompertz model can be directly estimated and interpretable, increasing the transparency of the effect of predictors on the outcome. Nevertheless, there are certain limitations inherent in this approach. First, deviations from the Gompertz mortality law emerge with old age (>80 years), leading to reduced fit and accuracy in estimating mortality risk^34^. Among our participants aged between 40–70 years old, however, the applicability of the Gompertz mortality law remains consistent. Next, our current approach assumes a linear combination of circadian parameters. The effects of circadian parameters on the rate of CosinorAge may vary or weigh differently with aging. In this study, we observed generally linear associations of circadian parameters with aging, likely due to our limited age range. For amplitude, we observed a relatively stable pattern until the age of 60, followed by a substantial decline. This phenomenon can be attributed to two potential explanations. First, a non-linear, age-related change in endogenous rhythmicity may lead to a significant alteration in circadian rhythms at age 60, as demonstrated by Rahman et al.^35^. Their study revealed stability in human plasma lipidome up to middle age, followed by a notable reduction in amplitude after reaching the age of 60. Second, this observation may be linked to the transition into retirement among the study participants. Studies have indicated that the transition to retirement can influence physical activity, sleep, and circadian rhythmicity^36^. Analyzing the distribution of participants across “Paid employment,” “Retired,” and “Unemployed” categories unveils a noteworthy surge in the percentage of participants classified as “Retired” between ages 55 and 60 (Supplementary Fig. 3). The distinction between MESOR and amplitude by employment status implies that the rhythm-adjusted activity level (e.g., MESOR) experiences a consistent decline with aging, while the magnitude of rhythmic change (e.g., amplitude) may be influenced by external factors such as employment status. Prior research has reported a relatively stable pattern followed by a temporary increase in MVPA during the transition to statutory retirement, succeeded by a subsequent decline in the post-retirement period, paralleling the observed pattern in this analysis^37^. Lastly, our inclusion of three parameters to describe circadian rhythmicity has shown robust predictive performance of longevity and healthspan, but additional information describing consistency, reliability, stability, or unique characteristics of an individual’s rhythmicity can contribute to more precise and tailored prediction. In future work, we aim to enhance the robustness of our findings by conducting secondary analyses to assess the consistency of daily rhythms at both individual and cohort levels. This process may involve leveraging longitudinal data, or it can also be accomplished with the current dataset comprising one week of wearable data. Existing research provides methodologies for effectively evaluating the consistency of circadian rhythms of activity, even when dealing with limited digital activity datasets^38,39^.

We confirmed the robustness and reliability of our approach through a rigorous development and validation process, incorporating external validation using an independent cohort from a disparate geographical region using a different wearable device. Our study is unique and provides preliminary evidence of a digital longevity biomarker, a step toward a scalable and easily accessible biomarker for aging. We also conducted several sensitivity analyses and demonstrated the consistency of results to the main analyses (Supplementary Tables 6–8). First, we excluded participants with a history of shift work and re-ran the analysis using the UK Biobank validation cohort. Second, we additionally adjusted for sleep disorders diagnosed prior to baseline assessment. Third, we included intradaily variability and interdaily stability as additional parameters to the regression models. Lastly, we excluded participants with less than 2 years of follow-up time to avoid reverse causation in the evaluation of disease incidences.

Our study possesses several strengths. First, we included a large sample of 80,000 participants with continuous 7-day physical activity data obtained in two nationwide population-level datasets, namely UKB and NHANES. Second, the robust predictive power of circadian rhythm-based age in terms of mortality, disease incidence risks, and healthspans underscores its clinical utility and potential for widespread implementations in the general public as well as healthcare professionals. Third, CosinorAge is an explainable and easy-to-interpret approach with a detailed characterization of underlying circadian factors influencing its prediction and advanced aging.

Nonetheless, we acknowledge certain limitations in our study. First, accelerometer data were collected for a limited period of 7 days. Extended and repeated measurements may be necessary to evaluate the reliability of the proposed approach. Second, time-varying patterns or inconsistent cycles may not fit well with the parametric cosinor model. To address this concern, we assessed rhythm regularity as part of our inclusion criteria and conducted a series of sensitivity analyses aimed at mitigating the effects of shift work, sleep disorders, and rhythm fragmentation and consistency. We found that these factors did not significantly confound our results. Next, it is worth noting that UKB participants tend to be healthier and predominantly of White race/ethnicity compared to the general UK population. To account for this, we included race/ethnicity and other sociodemographic factors in our models. Lastly, UKB and NHANES utilized different accelerometer placement instructions, with UKB placing the accelerometer on the dominant wrist of participants, while NHANES used the non-dominant wrist. A previous study has demonstrated that this discrepancy in placement instructions is minimal and accelerometer measurements from the non-dominant are largely compatible with those from the dominant wrist^40^.

In conclusion, we utilized 7-day wearable accelerometry data to characterize circadian rhythmicity and predict an individual’s longevity and healthspan, called CosinorAge. CosinorAge predicts mortality and morbidity risks, and strongly indicates functional performance decline even after adjusting for potential covariates. These findings are consistent in an independent, external cohort. Our research sheds lights on digital assessments of aging and healthspan based on wearable-derived circadian characteristics. Our study introduces an innovative and economic method that in the future might allow individuals, ranging from those with particular medical conditions to those in good health interested in disease prevention, to monitor their aging process. Future research is warranted to incorporate repeated, long-term measurements of circadian rhythms using digital wearables to examine the longitudinal trajectory of the aging process in real-life settings, involving diverse prospective cohorts utilizing various devices.

## Methods

### Data sources and study population

This study primarily focuses on two large, population-level datasets: the UK Biobank (UKB) and the US National Health and Nutrition Examination Survey (NHANES)^41,42^. UKB is a prospective cohort study including over 500,000 adults aged 40–69 years recruited between 2006 and 2010, which received ethics approval from the North West Multi-centre Research Ethics Committee (16/NW/0274). This research has been conducted using the UK Biobank Resource under Application Number 102250. Mortality records were linked to the UKB dataset from NHS Digital (England and Wales) and the Information and Statistics Division (Scotland). Follow-up periods were from the start date of accelerometer use to the registered date of death for the deceased or to the censoring date (November 30, 2022) for those who survived. We used the UKB data to train and develop an age estimation approach.

Additionally, we used NHANES data as an external validation set to assess the transportability of the predictive model to settings beyond those considered during its development. External validation cohort involves an independent sample from a comparable population, considering factors such as inclusion and exclusion criteria, while allowing for temporal or geographic differences, which supports our choice of the NHANES data^43–45^. The NHANES is a nationwide cross-sectional survey conducted by the Centers for Disease Control and Prevention. We included data from the 2011–2014 cycle, for which 7-day accelerometer data were available. The National Center for Health Statistics Ethics Review Board approved the NHANES study protocols (NCHS IRB/ERB Protocol Number: #2011–17). We obtained the mortality information from a publicly available file from the National Centre for Health Statistics with certified death records from the National Death Index. The follow-up periods refer to the time from the baseline assessment to the registered date of death for the deceased or the end of the follow-up period (December 31, 2019) for those who survived.

**Fig. 7:**
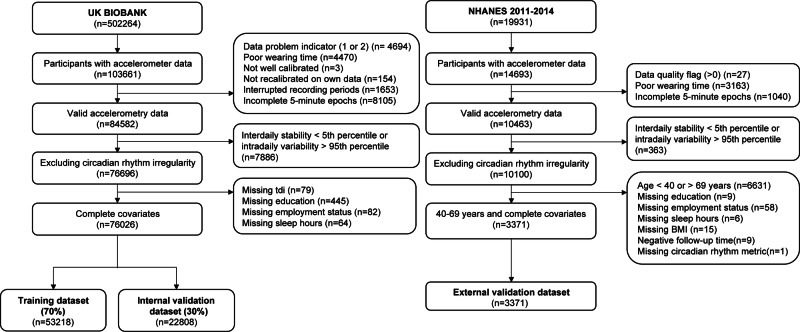
Flowchart for inclusion of study participants. *NHANES* US National Health and Nutrition Examination Survey.

For the present study, we included a total of 80,353 participants aged 40–69 years with confirmed mortality status, valid accelerometer recordings, regular circadian rhythmicity, and complete covariate information (Fig. 7). Participants in UKB and NHANES provided written informed consent. All methods were performed in accordance with the Declaration of Helsinki.

### Data processing of wearable-derived accelerometer activity data

Between 2013 and 2015, 236,519 UKB participants were randomly selected and invited to participate in the accelerometer assessment, of which 103,695 submitted data^28^. Accelerometer recordings from 103,661 participants were accessible at the time of analysis. Participants wore an Axivity AX3 (Axivity Ltd, Newcastle, UK) triaxial accelerometer on the wrist for 7 consecutive 24-hour periods. The sensor captured raw acceleration at 100 Hz resolution. A movement-intensity signal was derived by calculating the Euclidean Norm Minus One (ENMO) metric, where one gravitational unit (1 g) is subtracted from the vector magnitude of acceleration in three axes, with all negative values set to 0^28^. Using ENMO values in 5-s epochs, we obtained average acceleration at the minute level for each participant. Based on the data quality metrics provided by the UKB accelerometer working group, we excluded *n* = 19,079 participants with unreliable accelerometry data if any of the following conditions were met: flagged by the UKB as having data problem; insufficient wear time (<72 h of wear time or no wear data in each 1 h period of the 24-hour cycle); unreliable or not well-calibrated data; and accelerometry data with a nonzero count of interrupted recording periods^28^. We further excluded participants with missing in any of the 5-minute epochs per day to ensure the continuous time series of accelerometry data over the course of 24-hours^18^.

In NHANES, 14,693 participants who were aged 6 years and older during the 2011–2012 cycle or aged 3 years and older during the 2013–2014 cycle wore an ActiGraph GT3X+ (Actigraph, Pensacola, FL) triaxial accelerometer on the wrist for 7 consecutive 24-hour periods collecting raw signals on the x, y, and z axes with a sampling rate of 80 Hz. We converted the raw accelerometry obtained from the ActiGraph device to ENMO values at a minute level using the *SummarizedActigraphy* R package. We excluded 4230 participants who had data quality issues, did not meet sufficient wear time (<16-hour of wear time per day or <4 wear days), or had missing in any of the 5-minute epochs in 24-hours^46,47^.

### Assessment of circadian regularity

To ensure a reliable and robust age estimation regarding circadian rhythmicity, we assessed the degree of rhythm regularity of each individual and excluded participants displaying extremely irregular or time-varying circadian patterns. Circadian regularity is assessed based on non-parametric measures of the day-to-day stability of circadian patterns (i.e., interdaily stability; IS) and rhythm fragmentation (i.e., intradaily variability; IV) computed using *nparACT* R package^48^. IS ranges from 0 to 1, with a low value indicating an unstable rhythm with poor synchronization to the 24-hour light-dark cycle. IV ranges from 0 to 2, with a higher value indicating a more fragmented circadian rhythm. After testing different cutoffs, we excluded 8249 participants (7886 from UKB and 363 from NHANES) with extremely inconsistent (IS value < 5th percentile) or fragmented (IV value > 95th percentile) circadian rhythms. The remaining 79,397 participants (76,026 from UKB and 3371 from NHANES) are included in the development and validation of the CosinorAge approach.

### Cosinor analysis

The wearable-derived accelerometry pattern indicates circadian rhythmicity, reflecting the timing and rhythmic fluctuations of physical activity, sleep, and rest-activity rhythm^49^. We characterized the circadian 24-hour cycle using cosinor analysis, which examines the degree of fit between the wearable data and a superposition of cosine functions, allowing us to calculate circadian parameters of MESOR, amplitude, and acrophase^50,51^. The cosinor model has been widely applied to analyze circadian markers based on cortisol, melatonin, and core body temperature^52,53^. A single component cosinor model is written as in Eq. (1):

1 $$Y\left(t\right)=M+A\,\cdot\, \cos \left(\frac{2\pi t}{\tau }+\varphi \right)+e\left(t\right)$$

where *Y(t)* refers to the accelerometry activity level at time t. *M* indicates the rhythm-adjusted average activity level, known as the Midline Estimating Statistic of Rhythm (MESOR). Amplitude (*A*) is a measure of half the extent of the variation within the cycle. Acrophase (*φ*) measures the time of the maximum activity level. Period (*τ*) is a duration of one cycle, and *e(t)* is the error term. The non-linear problem of fitting a cosine function is reduced to a problem with linear parameters by applying the least squares methods as follows, thus allowing to extract circadian parameters, as depicted in Eq. (2):

2 $$Y\left(t\right)=M+\beta x+\gamma z+e\left(t\right)$$

$${\rm{where}}\,\beta =A\cos \left(\varphi \right){\rm{;}}\,x=\cos \left(\frac{2\pi t}{\tau }\right){\rm{;}}\,\gamma =-A\sin \left(\varphi \right){\rm{;}}\,z=\sin \left(\frac{2\pi t}{\tau }\right){\rm{;}}$$

The best fitting period of the cosinor model of 24-hour was estimated using iterative cosinor procedures. We used *cosinor*, *cosinor2*, and *card* R packages for computation. The acrophase was corrected using the method proposed by Bingham et al.^54^.

### Development and validation of CosinorAge

To avoid over-fitting, we deployed a 70:30 split of training/internal validation set of UK Biobank data (*n* = 76,026) to develop and validate new age approach. In addition, NHANES data (*n* = 3371) served as an external validation set. CosinorAge is based on the multivariate analysis of hazard risks proposed by PhenoAge. In the training data, we estimated the 5-year mortality risk of an individual from a parametric proportional hazard model based on the Gompertz distribution using circadian rhythm parameters and age. We then fit a separate univariate Gompertz proportional hazard model using chronological age. CosinorAge was obtained by equating these two models and solving for age. More details can be found in Supplementary Methods.

In validating the CosinorAge approach, we quantified the rate of aging by calculating ‘CosinorAgeAdvance’, which represents the difference between the estimated biological age and actual chronological age of an individual. We then assessed the associations between CosinorAge and a range of longevity outcomes, including all-cause and cause-specific mortality risks, prospective risks of developing aging-related diseases, measures of aging-related functional performance, and established biological age estimators.

### Association between CosinorAge and mortality and incident disease risks

We evaluated associations of CosinorAge with all-cause, age-related, and disease-specific mortalities attributed to CVD, cancer, diabetes, neurodegenerative, and chronic respiratory diseases in UKB and NHANES validation cohorts (Supplementary Table 9). Aging-related mortality is defined as death resulting from any of the following diseases: CVD, cancer, diabetes, neurodegenerative, or chronic respiratory diseases. Participants with prevalent diseases corresponding to the related disease-specific mortalities were excluded from the relevant analyses.

In the assessments of CosinorAge with the future risks of aging-related diseases, we included 14,658 participants from the UKB validation cohort who were free from hypertension, CVD, cancer, diabetes, neurodegenerative, and chronic respiratory diseases at baseline. We identified incident and prevalent diseases considering self-reported touchscreen questionnaires, self-reported interviews, all instances of hospital admission and death, and the cancer registry (for cancer incidences only) (Supplementary Table 10). In self-reported interviews, we defined incident cases if the age of diagnosis was equal to or greater than the age at the start date of accelerometer use. In the hospital, death, and cancer registry records, we defined incident cases when the onset date of illness was later than the start date of accelerometer use. Otherwise, cases were classified as prevalent. The first incidence was defined as the first occurrence of any of the following diseases: hypertension, CVD, cancer, diabetes, neurodegenerative, and chronic respiratory diseases.

We obtained the hazard ratios (HRs) and corresponding 95% CIs using multivariable Cox proportional hazards model adjusting for age, sex, ethnicity/race, Townsend deprivation index (UKB only), baseline assessment center (UKB only), education, employment, body mass index, smoking status, alcohol consumption, and comorbidities (mortality models) with *survival* R package. Diabetes- and chronic respiratory disease-specific mortalities were adjusted for age to achieve model convergence. To account for the complex survey design and produce representative estimates of the US population, we applied four-year survey weights to statistical procedures in NHANES sample using *survey* R package^55^. The proportional-hazards assumption was formally tested using Schoenfeld residuals, and no significant violation was found. To account for multiple testing, we applied the Bonferroni correction and considered a two-sided *p* < 0.007 (i.e., 0.05/7) as statistical significance.

### Association between CosinorAge and aging-related functional performance

We further examined the associations between CosinorAge and various aging-related functional performance measures, including self-rated health, grip strength, and health-related quality of life (HRQoL) scores. Participants’ self-rated health was assessed using the question “In general how would you rate your overall health?”. Response options included “Poor”, “Fair”, “Good”, and “Excellent” in UK Biobank and “Poor”, “Fair”, “Good”, “Very Good”, and “Excellent” in NHANES. Grip strength was measured using a hand dynamometer, and we used the maximum reading across measurements in UK Biobank and the maximum reading for the dominant hand in NHANES. In assessments of HRQoL, we implemented the European Quality of Life-5 Dimensions 5-levels (EQ-5D) in the UK Biobank and the activities of daily living (ADL) in NHANES. Both EQ-5D and ADL have been validated in several populations. EQ-5D evaluated five dimensions, including mobility, self-care, usual activities, pain and discomfort, and anxiety and depression, and each dimension has five levels of response (from ‘no problems’ to ‘extreme problems’)^56^. Participants self-reported a five-dimensional health descriptive system and self-rated overall health using the EuroQol visual analogue scale^56^. The utility score is calculated according to the EQ-5D Guide, with higher scores indicating better HRQoL. In the NHANES, functional abilities were assessed through ADL score from 19 items^57^. The responses to these items were graded on a 4-point Likert-type scale with a score ranging from one to four representing the level of difficulty in performing various tasks (1 = no difficulty, 2 = some difficulty, 3 = much difficulty, and 4 = unable to do)^57^. The ADL scale ranges from 0 to 76, with lower scores indicating better functional performance.

We used multivariable generalized linear models to assess the association between CosinorAge advancement and each of the functional outcomes using the *stats* R package. CosinoarAgeAdvance and outcomes were standardized with a mean value of 0 and standard deviation (SD) of 1 in continuous scale and to quartile groups to facilitate the interpretation of results. Dose-response curves for associations with outcomes were assessed using *plotPCS* R package by restricted cubic spline regression models controlling for all covariates previously mentioned, with the 25th, 50th, and 75th percentiles of CosinorAgeAdvance selected as knots. We account for complex survey design in NHANES.

### Association between CosinorAge and established biological ages

We tested the correlations of CosinorAge with two previously established clinical-parameter biological ages, namely Klemera-Doubal biological age (KDM BA) and phenotypic age (PhenoAge). KDM BA and PhenoAge, both validated across multi-ethnic cohorts of adults to predict disease, disability, and mortality, are widely recognized as reliable estimators of biological aging^58,59^. Briefly, KDM BA was computed from forced expiratory volume in one second, systolic blood pressure, albumin, alkaline phosphatase, blood urea nitrogen, creatinine, C-reactive protein, glycated hemoglobin, and total cholesterol; PhenoAge was computed from chronologicla age and nine blood chemistries including albumin, alkaline phosphatase, creatinine, C-reactive protein, glucose, mean cell volume, red cell distribution width, white blood cell count, and lymphocyte proportion^3,60^. We used *BioAge* R package to train and compute KDM BA and PhenoAge measures by sex^61^. Pearson’s correlation coefficients were used, and any observations with missing values were excluded from this analysis.

### Development of PA age (physical activity-based age)

To compute PA Age, we obtained the MVPA information through the UK Biobank (cf. Covariates). The mean MVPA duration (hours/day) during the monitoring period was calculated at the participant level. In the training data, we estimated the 5-year mortality risk from a Gompertz proportional hazard regression using MVPA duration and age, which was then converted to PA Age. We quantified the rate of aging, denoted as ‘PA Age Advance’, by taking a difference between PA Age and actual chronological age.

### Predictive performance metrics

To assess and compare the predictive performance of CosinorAge and PA Age regarding mortality and disease incidence outcomes, we computed Harrel’s concordance index (C-index) and Akaike information criteria (AIC) metric of the models using the internal validation set^62,63^. The C-index is an extension of the area under the receiver operating characteristic curve to survival analysis accounting for censoring^64^. The C-index quantifies the proportion of correctly ordered pairs among all comparable pairs, and its 95% CI is calculated based on 1000 bootstrap samples. The AIC is an information-based criterion assessing the model’s fit to data^65^. Higher C-index and lower AIC values deem superior discrimination ability. To compare the associations between the rates of CosinorAge vs. PA Age and outcomes, we categorized individuals into fast aging (CosinorAgeAdvance > 0 or PA Age Advance > 0) or slow aging (CosinorAgeAdvance ≤ 0 or PA Age Advance ≤ 0) groups. We depicted the cumulative risks by the rate of aging in Kaplan–Meier plots. Subsequently, we fitted multivariable Cox regression hazards models to obtain the HR and 95% CI for each outcome adjusting for covariates.

### Sleep disturbance

We employed two distinct definitions for sleep disturbance to ensure accurate assessments^66,67^. First, we defined sleep disturbance based on sleepiness/insomnia. Participants were asked: “Do you have trouble falling asleep at night or do you wake up in the middle of the night?” with response options “Never/rarely”, “Sometimes”, “Usually”, or “Prefer not to answer”. Participants were classified as experiencing sleep disturbance if they answered “Usually” to the question. Second, we broadened the first definition by incorporating snoring and daytime dozing as additional traits. The snoring question was assessed with: “Does your partner or a close relative or friend complain about your snoring?” with response options “Yes”, “No”, or “Prefer not to answer”. Participants were classified as experiencing snoring if they answered “Yes” to the question. Daytime dozing/sleeping was assessed by asking: “How likely are you to doze off or fall asleep during the daytime when you don’t mean to? (e.g. when working, reading or driving)” with response options “Never/rarely”, “Sometimes”, “Often”, “All of the time” or “Prefer not to answer”. Participants were classified as experiencing daytime dozing if they answered “Often” or “All of the time”. Participants were classified as experiencing sleep disturbance if they reported one or more symptoms of insomnia, snoring, or daytime dozing.

### Covariates

We obtained additional information on characteristics a priori that would be associated with circadian rhythm and aging based on previous research^17^^,27^: age, sex, ethnicity/race (white/non-white), Townsend deprivation index (UKB only), baseline assessment center (UKB only), education (college or above/high school or equivalent/less than high school), body mass index (normal or underweight: <25 kg/m^2^, overweight: 25–29.9 kg/m^2^, obese: ≥30 kg/m^2^), smoking status (never/previous/current), alcohol consumption (not current/<3 times a week/≥3 times a week), employment (paid employment/unpaid), history of shift work (UKB only), and comorbidities. Body-mass index was calculated from measurements of height (m) and weight (kg) using weight divided by height^2^. We derived physical activity durations from accelerometry data, including sleep, sedentary, light intensity activity (LPA), and moderate-to-vigorous intensity activity (MVPA). In UKB, an established machine-learning algorithm is used for the classification of movement behaviors based on metabolic equivalents of task (METs)^68^. In brief, this algorithm extracted time and frequency domain features for non-overlapping 30-s time window and uses random forests with a hidden Markov model for smoothing to predict behaviors. Time spent per day were reported for MVPA (≥3 METs), LPA (1.5–3 METs), sedentary behaviors (<1.5 METs), and sleep (non-waking behavior)^68^. In NHANES, we applied the standard cut points to the converted minute-level ENMO values to classify physical activities. Daily time spent in LPA and MVPA were derived from cut-points of 45–100 mg and >100 mg, respectively. Daily time spent in sedentary behavior was obtained by subtracting the time spent <45 mg from the participants’ reported duration spent sleeping per night. The average duration of sleep, sedentary, LPA, and MVPA are calculated for each individual (hours/day). All analysis was performed using R software 4.3.1 and RStudio 2023.06.1.

### Supplementary information

Supplementary Information

## Data Availability

The UK Biobank data are available for research purposes by application. The NHANES data are publicly available from the Centers for Disease Control and Prevention website.

## Code availability

The codes that support the findings of this study are available upon request addressed to the corresponding author.
