# Supplementary material for: Circadian rhythm analysis using wearable-based accelerometry as a digital biomarker of aging and healthspan
Source: NPJ Digit Med. 2024 Jun 4;7:146. doi: 10.1038/s41746-024-01111-x (PMC11150246; doi:10.1038/s41746-024-01111-x)
Supplement: Supplementary file 1 — Supplementary Information [file 41746_2024_1111_MOESM1_ESM.pdf]

**SUPPLEMENTAL MATERIAL**

# Circadian Rhythm Analysis Using Wearable-Based Accelerometry as a Digital Biomarker of Aging and Healthspan

Jinjoo Shim[1*], Elgar Fleisch[1,2], Filipe Barata[1]

[1]Centre for Digital Health Interventions, ETH Zurich, Zurich, Switzerland;
[2]Centre for Digital Health Interventions, University of St. Gallen, St. Gallen, Switzerland

***Corresponding author:**
Jinjoo Shim
Centre for Digital Health Interventions
ETH Zurich
Weinbergstrasse 56/58
8092 Zurich, Switzerland
jshim@ethz.ch

**Table of Contents**

**Supplementary Fig. 1** Circadian rhythm parameters with chronological age.

**Supplementary Table 1.** Gompertz proportional hazards regression models for CosinorAge using the training dataset.

**Supplementary Table 2.** Sex-stratified associations between CosinorAge advancement and mortality risks.

**Supplementary Table 3.** Sex-stratified associations between CosinorAge advancement and disease incidences.

**Supplementary Table 4.** Sex-stratified associations between CosinorAge advancement and age-related functional performances.

**Supplementary Fig. 2** Associations between CosinorAge and established biological aging measures in validation cohorts stratified by sex.

**Supplementary Table 5.** Sex-stratified age-dependent variations in circadian rhythm metrics by CosinorAge advancement.

**Supplementary Table 6.** Sensitivity analyses of associations between CosinorAge advancement and mortality risks.

**Supplementary Table 7.** Sensitivity analyses of associations between CosinorAge advancement and disease incidences.

**Supplementary Table 8.** Sensitivity analyses of associations between CosinorAge advancement and age-related functional performances.

**Supplementary Fig. 3** Distribution of employment status in UK Biobank cohort.

**Supplementary Table 9.** International Classification of Diseases (ICD) codes used for identifying cause-specific mortality.

**Supplementary Table 10.** Codes used for identifying prevalent and incident diseases in UK Biobank.

**Supplementary Methods.** Derivation of CosinorAge

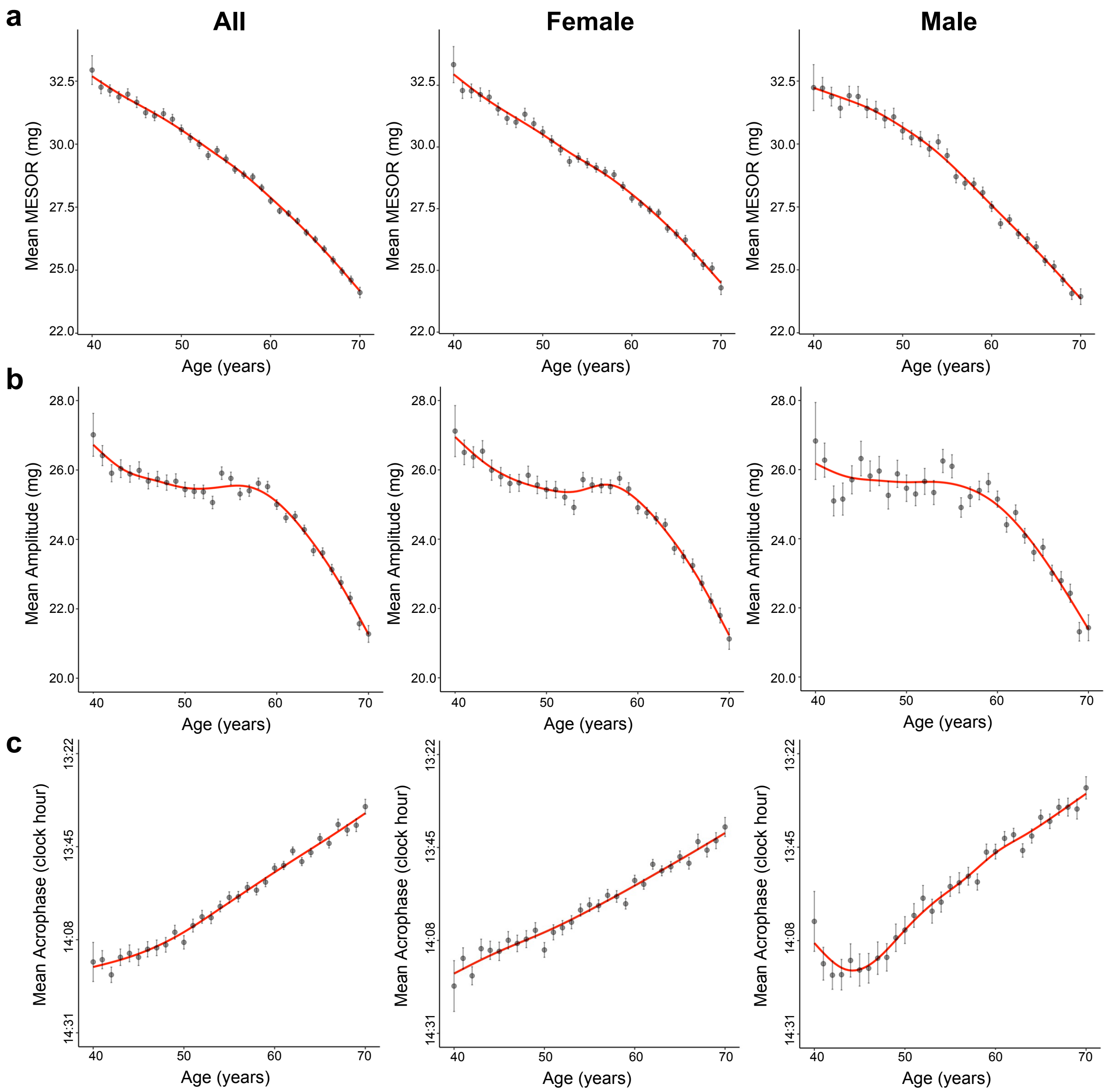


**Supplementary Fig. 1** Circadian rhythm parameters with chronological age. **a** Midline Estimating Statistic of Rhythm (MESOR). **b** Amplitude. **c** Acrophase. Mean values and the standard errors are shown for individuals categorized by chronological age.

**Supplementary Table 1.** Gompertz proportional hazards regression models for CosinorAge using the training dataset.

| Variable | All | Female | Male |
|---|---|---|---|
| MESOR (mg) | -0.032 | -0.026 | -0.024 |
| Amplitude (mg) | -0.020 | -0.022 | -0.031 |
| Acrophase (radiant) | -0.017 | -0.132 | 0.009 |
| Chronological age (years) | 0.100 | 0.088 | 0.102 |
| Shape | 0.015 | 0.013 | 0.014 |
| Rate | -13.367 | -13.285 | -13.017 |

$$\text{CosinorAge}_{\text{all}} = 133.599 + \frac{\ln[-0.015 * \ln(1 - mortality\ risk)]}{0.112}$$

$$\text{mortality score (MS)} = 1 - e^{\frac{-(e^{60\gamma}-1)(e^{bx})}{\gamma}}$$

$$\gamma = 0.015$$

$$bx = -13.367 - 0.032 * MESOR - 0.02 * amplitude - 0.017 * acrophase + 0.1 * chronological\ age$$

**Supplementary Table 2.** Sex-stratified associations between CosinorAge advancement and mortality risks.

| Mortality | Male | | Female | |
|---|---|---|---|---|
| | Events/ Participants | HR (95% CI) | Events/ Participants | HR (95% CI) |
| **Internal Validation (UKB)** | | | | |
| All-cause | 473/9045 | 1.07 (1.04, 1.10)* | 395/13760 | 1.11 (1.07, 1.14)* |
| Aging-related | 403/8975 | 1.07 (1.03, 1.10)* | 345/13710 | 1.11 (1.06, 1.15)* |
| CVD | 87/8630 | 1.13 (1.05, 1.22)* | 55/13393 | 1.03 (0.94, 1.13) |
| Cancer | 197/7713 | 1.06 (1.01, 1.11)* | 165/11569 | 1.08 (1.03, 1.14)* |
| Diabetes | 14/8212 | 1.37 (1.11, 1.68)* | 3/13029 | 1.27 (0.84, 1.93) |
| Neurodegenerative | 34/8599 | 1.25 (1.10, 1.42)* | 27/13371 | 1.33 (1.13, 1.55)* |
| Respiratory | 14/8474 | 1.17 (0.97, 1.40) | 8/13209 | 1.29 (0.99, 1.69) |
| **External Validation (NHANES)** | | | | |
| All-cause | 113/1528 | 1.14 (1.06, 1.23)* | 86/1842 | 1.15 (1.06, 1.25)* |
| Aging-related | 75/1490 | 1.13 (1.02, 1.25)* | 61/1817 | 1.11 (1.01, 1.22)* |

Cox proportional hazard regression models were adjusted for age, ethnicity/race, Townsend deprivation index (UKB only), baseline assessment center (UKB only), education, employment, body mass index, smoking status, alcohol consumption, and comorbidities. Diabetes- and chronic respiratory disease-specific mortalities were adjusted for age to achieve model convergence. We account for complex survey design in NHANES. Participants with prevalent diseases corresponding to the related disease-specific mortalities were excluded from the relevant analyses. Aging-related mortality is defined as death resulting from any of the following diseases: CVD, cancer, diabetes, neurodegenerative (UKB only), and chronic respiratory diseases. *$p<0.05$.

**Supplementary Table 3.** Sex-stratified associations between CosinorAge advancement and disease incidences.

| Disease Incidence | Male | | Female | |
|---|---|---|---|---|
| | Events/ Participants | HR (95% CI) | Events/ Participants | HR (95% CI) |
| First incidence | 1510/5552 | 1.02 (1.00, 1.03)* | 1690/9106 | 1.04 (1.03, 1.06)* |
| Hypertension | 632/5552 | 1.04 (1.01, 1.06)* | 691/9106 | 1.04 (1.01, 1.06)* |
| CVD | 423/5552 | 1.03 (1.00, 1.06)* | 282/9106 | 1.03 (0.99, 1.07) |
| Cancer | 746/5552 | 1.01 (0.99, 1.03) | 867/9106 | 1.02 (1.00, 1.04) |
| Diabetes | 116/5552 | 1.11 (1.05, 1.18)* | 95/9106 | 1.16 (1.08, 1.25)* |
| Neurodegenerative | 63/5552 | 1.14 (0.97, 1.12) | 62/9106 | 1.29 (1.17, 1.42)* |
| Respiratory | 105/5552 | 1.03 (0.97, 1.08) | 134/9106 | 1.06 (1.00, 1.12)* |

Cox proportional hazard regression models were adjusted for age, ethnicity/race, Townsend deprivation index, baseline assessment center, education, employment, body mass index, smoking status, and alcohol consumption. Diabetes- and chronic respiratory disease-specific incidences were adjusted for age to achieve model convergence. Participants with prevalent diseases were excluded from the analyses. First incidence was defined as the first occurrence of any of the aforementioned diseases. *p<0.05.

**Supplementary Table 4.** Sex-stratified associations between CosinorAge advancement and age-related functional performances.

| | Male - Internal Validation (UKB) | | | | | | Female - Internal Validation (UKB) | | | | | |
|---|---|---|---|---|---|---|---|---|---|---|---|---|
| **CosinorAge** | **Self-Rated Health** (higher = poorer health) | | **Grip Strength** (lower = poorer strength) | | **EQ-5D** (lower = poorer HRQoL) | | **Self-Rated Health** (higher = poorer health) | | **Grip Strength** (lower = poorer strength) | | **EQ-5D** (lower = poorer HRQoL) | |
| | Coefficients (SE) | p-value | Coefficients (SE) | p-value | Coefficients (SE) | p-value | Coefficients (SE) | p-value | Coefficients (SE) | p-value | Coefficients (SE) | p-value |
| CAA (Continuous) | 0.09 (0.009) | <0.001* | 0.002 (0.008) | 0.746 | -0.02 (0.011) | <0.03* | 0.10 (0.008) | <0.001* | -0.03 (0.005) | <0.001* | -0.07 (0.011) | <0.001* |
| **CAA (Quartiles) (Q1: Reference)** | | | | | | | | | | | | |
| Q2 | 0.09 (0.027) | <0.001* | 0.02 (0.023) | 0.778 | -0.03 (0.033) | 0.349 | 0.08 (0.021) | <0.001* | -0.04 (0.013) | 0.003* | -0.06 (0.027) | 0.04* |
| Q3 | 0.13 (0.027) | <0.001* | 0.03 (0.022) | 0.175 | -0.04 (0.033) | 0.203 | 0.12 (0.022) | <0.001* | -0.04 (0.013) | 0.006* | -0.12 (0.028) | <0.001* |
| Q4 | 0.23 (0.027) | <0.001* | 0.01 (0.022) | 0.789 | -0.06 (0.033) | 0.064 | 0.28 (0.023) | <0.001* | -0.06 (0.013) | <0.001* | -0.17 (0.029) | <0.001* |
| | **Male - External Validation (NHANES)** | | | | | | **Female - External Validation (NHANES)** | | | | | |
| **CosinorAge** | **Self-Rated Health** | | **Grip Strength** | | **ADL** (higher = poorer HRQoL) | | **Self-Rated Health** | | **Grip Strength** | | **ADL** (higher = poorer HRQoL) | |
| | Coefficients (SE) | p-value | Coefficients (SE) | p-value | Coefficients (SE) | p-value | Coefficients (SE) | p-value | Coefficients (SE) | p-value | Coefficients (SE) | p-value |
| CAA (Continuous) | 0.08 (0.037) | 0.052 | 0.02 (0.036) | 0.628 | 0.07 (0.017) | <0.001* | 0.13 (0.034) | <0.001* | 0.02 (0.020) | 0.294 | 0.12 (0.028) | <0.001* |
| **CAA (Quartiles) (Q1: Reference)** | | | | | | | | | | | | |
| Q2 | 0.03 (0.067) | 0.670 | -0.01 (0.063) | 0.838 | -0.03 (0.062) | 0.632 | 0.13 (0.104) | 0.235 | -0.01 (0.055) | 0.795 | 0.08 (0.065) | 0.242 |
| Q3 | 0.08 (0.097) | 0.421 | 0.08 (0.058) | 0.199 | 0.11 (0.074) | 0.140 | 0.12 (0.090) | 0.211 | 0.09 (0.046) | 0.053 | 0.05 (0.061) | 0.391 |
| Q4 | 0.34 (0.111) | 0.006* | 0.01 (0.097) | 0.915 | 0.18 (0.055) | 0.004* | 0.32 (0.101) | 0.005* | 0.04 (0.059) | 0.519 | 0.27 (0.072) | 0.001* |

Generalized linear models were adjusted for age, ethnicity/race, Townsend deprivation index (UKB only), baseline assessment center (UKB only), education, employment, body mass index, smoking status, alcohol consumption, and comorbidities. We account for complex survey design in NHANES. *CAA* CosinorAgeAdvance, *SE* standard errors, *EQ-5D* European Quality of Life-5 Dimensions 5-levels*, ADL* Activities of Daily Living, *HRQoL* Health-related quality of life. *p values < 0.05. We standardized the CAAs and outcomes to have a mean value of 0 and a standard deviation of 1.

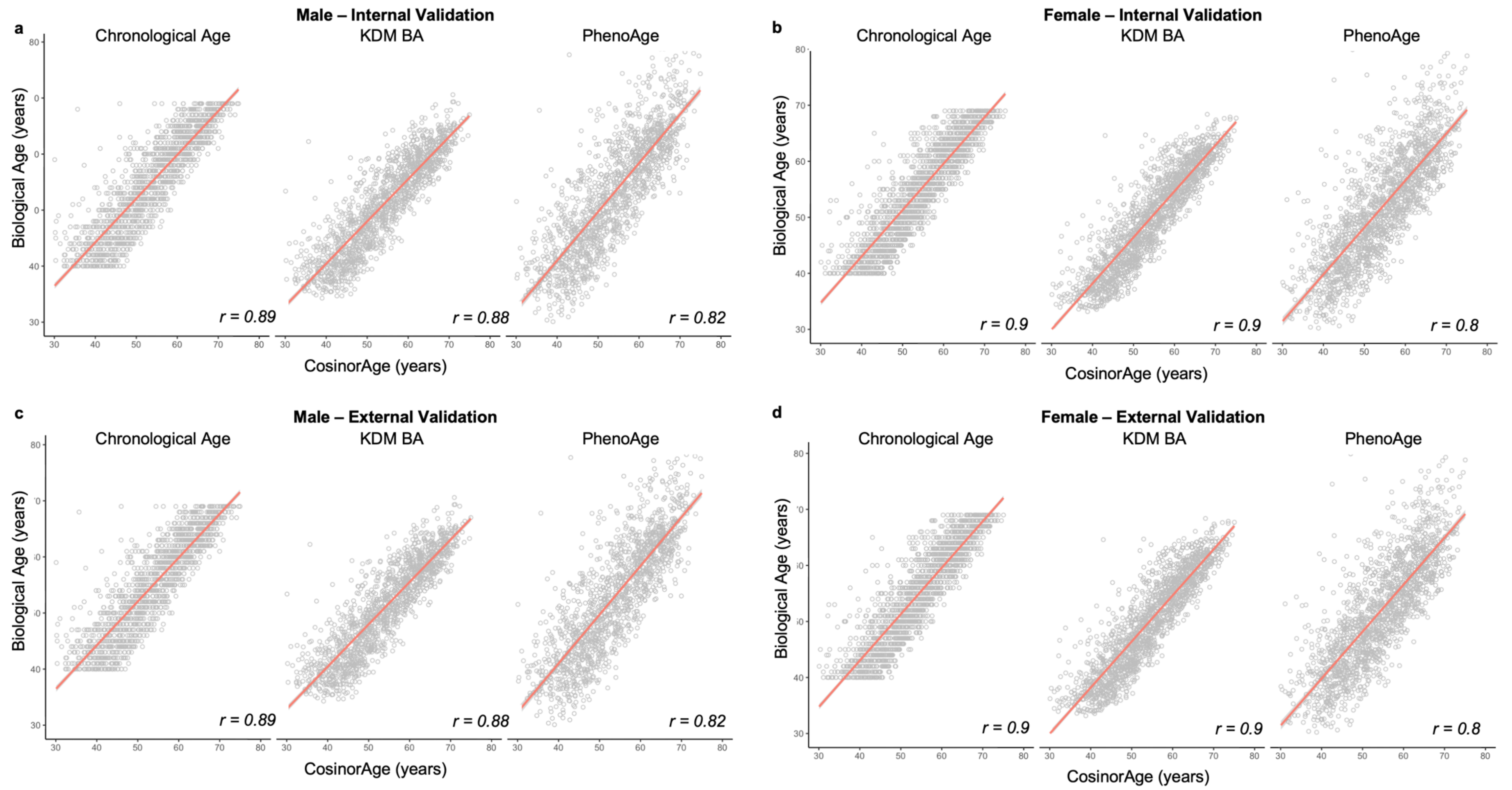


**Supplementary Fig. 2** Associations between CosinorAge and established biological aging measures in validation cohorts stratified by sex. **a,b** Correlations with chronological age, Klemera-Doubal biological age (KDM BA), and phenotypic age (PhenoAge) in the UKB internal validation cohort stratified by sex. **c,d** Correlations with chronological age, KDM BA, and PhenoAge among females in the NHANES external validation cohort stratified by sex. *r* Pearson's correlation coefficient.

**Supplementary Table 5.** Sex-stratified age-dependent variations in circadian rhythm metrics by CosinorAge advancement.

| Male - Internal Validation (UKB) | | | | | | | | | | | | |
|---|---|---|---|---|---|---|---|---|---|---|---|---|
| | MESOR (mg) | | | | Amplitude (mg) | | | | Acrophase (clock hour) | | | |
| **Age groups (years)** | **<45** | **46-55** | **56-65** | **>65** | **<45** | **46-55** | **56-65** | **>65** | **<45** | **46-55** | **56-65** | **>65** |
| Fast agers | 24.1 | 21.9 | 19.2 | 17.9 | 17.1 | 16.2 | 15.5 | 14.4 | 14:24 | 14:15 | 13:52 | 13:36 |
| Slow agers | 42.1 | 39.8 | 35.5 | 33.5 | 38.9 | 36.8 | 34.6 | 32.6 | 13:57 | 13:50 | 13:38 | 13:31 |
| Difference (Fast-Slow) | -18.0 | -17.9 | -16.3 | -15.6 | -21.8 | -20.6 | -19.1 | -18.2 | +27 minutes | +25 minutes | +14 minutes | +5 minutes |
| **Female - Internal Validation (UKB)** | | | | | | | | | | | | |
| | **MESOR (mg)** | | | | **Amplitude (mg)** | | | | **Acrophase (clock hour)** | | | |
| **Age groups (years)** | **<45** | **46-55** | **56-65** | **>65** | **<45** | **46-55** | **56-65** | **>65** | **<45** | **46-55** | **56-65** | **>65** |
| Fast agers | 23.7 | 22.2 | 19.5 | 17.8 | 18.0 | 16.9 | 15.5 | 14.5 | 14:15 | 14:10 | 14:01 | 13:54 |
| Slow agers | 41.7 | 39.0 | 35.5 | 33.6 | 37.1 | 35.3 | 33.2 | 30.9 | 13:54 | 13:57 | 13:45 | 13:40 |
| Difference (Fast-Slow) | -18.0 | -16.8 | -16.0 | -15.8 | -19.1 | -18.4 | -17.7 | -16.4 | +21 minutes | +13 minutes | +16 minutes | +14 minutes |

**Supplementary Table 6.** Sensitivity analyses of associations between CosinorAge advancement and mortality risks.

| Mortality | Model 1 | | Model 2 | | Model 3 | |
|---|---|---|---|---|---|---|
| | Events/ Participants | HR (95% CI) | Events/ Participants | HR (95% CI) | Events/ Participants | HR (95% CI) |
| **Internal Validation (UKB)** | | | | | | |
| All-cause | 827/21084 | 1.09 (1.06, 1.11)* | 868/22805 | 1.08 (1.06, 1.11)* | 868/22797 | 1.09 (1.06, 1.12)* |
| Aging-related | 711/20968 | 1.08 (1.06, 1.11)* | 748/22685 | 1.08 (1.06, 1.11)* | 748/22677 | 1.09 (1.06, 1.12)* |
| CVD | 135/10342 | 1.10 (1.04, 1.17)* | 142/22023 | 1.09 (1.03, 1.16)* | 142/22015 | 1.08 (1.02, 1.15)* |
| Cancer | 340/17745 | 1.07 (1.03, 1.11)* | 362/19282 | 1.07 (1.04, 1.11)* | 362/19274 | 1.07 (1.04, 1.11)* |
| Diabetes | 15/19615 | 1.15 (0.96, 1.37) | 17/21241 | 1.18 (0.99, 1.40) | 17/21233 | 1.17 (0.98, 1.39) |
| Neurodegenerative | 59/20290 | 1.28 (1.16, 1.42)* | 61/21970 | 1.28 (1.16, 1.42)* | 61/21962 | 1.36 (1.22, 1.51)* |
| Respiratory | 22/20030 | 1.12 (0.96, 1.30) | 22/21683 | 1.11 (0.96, 1.30) | 22/21675 | 1.07 (0.92, 1.26) |
| **External Validation (NHANES)** | | | | | | |
| All-cause | - | - | 199/3370 | 1.11 (1.05, 1.18)* | 199/3370 | 1.10 (1.04, 1.16)* |
| Aging-related | - | - | 136/3307 | 1.11 (1.03, 1.18)* | 136/3307 | 1.09 (1.03, 1.15)* |

Cox proportional hazard regression models were adjusted for age, ethnicity/race, Townsend deprivation index (UKB only), baseline assessment center (UKB only), education, employment, body mass index, smoking status, alcohol consumption, and comorbidities. Diabetes- and chronic respiratory disease-specific mortalities were adjusted for age to achieve model convergence. We account for complex survey design in NHANES. Participants with prevalent diseases corresponding to the related disease-specific mortalities were excluded from the relevant analyses. Aging-related mortality is defined as death resulting from any of the following diseases: CVD, cancer, diabetes, neurodegenerative (UKB only), and chronic respiratory diseases. *p<0.05. **Model 1** excluded participants with shift work history (UKB only). **Model 2** additionally adjusted for sleep disorders. **Model 3** additionally adjusted for circadian rhythm fragmentation and stability.

**Supplementary Table 7.** Sensitivity analyses of associations between CosinorAge advancement and disease incidences.

| Disease Incidence | Model 1 Events/ Participants | Model 1 HR (95% CI) | Model 2 Events/ Participants | Model 2 HR (95% CI) | Model 3 Events/ Participants | Model 3 HR (95% CI) | Model 4 Events/ Participants | Model 4 HR (95% CI) |
|---|---|---|---|---|---|---|---|---|
| First incidence | 2875/13447 | 1.03 (1.02, 1.04)* | 3200/14658 | 1.02 (1.01, 1.04)* | 3200/14650 | 1.02 (1.01, 1.04)* | 3165/14618 | 1.02 (1.01, 1.04)* |
| Hypertension | 1213/13447 | 1.04 (1.02, 1.15)* | 1323/14658 | 1.03 (1.01, 1.05)* | 1323/14650 | 1.03 (1.01, 1.05)* | 1315/14618 | 1.03 (1.01, 1.05)* |
| CVD | 649/13447 | 1.03 (1.00, 1.05)* | 705/14658 | 1.02 (1.00, 1.05)* | 705/14650 | 1.02 (1.00, 1.05)* | 695/14618 | 1.02 (1.00, 1.05)* |
| Cancer | 1513/13447 | 1.01 (0.99, 1.02) | 1613/14658 | 1.01 (0.99, 1.03) | 16113/14650 | 1.01 (0.99, 1.03) | 1587/14618 | 1.01 (0.99, 1.03) |
| Diabetes | 194/13447 | 1.12 (1.07, 1.18)* | 211/14658 | 1.10 (1.05, 1.15)* | 211/14650 | 1.09 (1.04, 1.14)* | 209/14618 | 1.10 (1.05, 1.15)* |
| Neurodegenerative | 119/13447 | 1.14 (1.07, 1.21)* | 125/14658 | 1.14 (1.07, 1.21)* | 125/14650 | 1.17 (1.09, 1.24)* | 124/14618 | 1.15 (1.08, 1.22)* |
| Respiratory | 223/13447 | 1.03 (0.99, 1.07) | 239/14658 | 1.03 (0.99, 1.07) | 239/14650 | 1.04 (0.99, 1.08) | 237/14618 | 1.03 (0.99, 1.07) |

Cox proportional hazard regression models were adjusted for age, ethnicity/race, Townsend deprivation index, baseline assessment center, education, employment, body mass index, smoking status, and alcohol consumption. Diabetes- and chronic respiratory disease-specific incidences were adjusted for age to achieve model convergence. Participants with prevalent diseases were excluded from the analyses. First incidence was defined as the first occurrence of any of the aforementioned diseases. *p<0.05. **Model 1** excluded participants with shift work history. **Model 2** additionally adjusted for sleep disorders. **Model 3** additionally adjusted for circadian rhythm stability and fragmentation. **Model 4** excluded participants with less than 2 years of follow-up time.

**Supplementary Table 8.** Sensitivity analyses of associations between CosinorAge advancement and age-related functional performances.

| Internal Validation (UKB): Model 1 | | | | | | |
|---|---|---|---|---|---|---|
| **CosinorAge** | **Self-Rated Health** | | **Grip Strength** | | **EQ-5D** | |
| | Coefficients (SE) | p-value | Coefficients (SE) | p-value | Coefficients (SE) | p-value |
| CAA (Continuous) | 0.09 (0.006) | <0.001* | -0.03 (0.006) | <0.001* | -0.05 (0.008) | <0.001* |
| **CAA (Quartiles) (Q1: Reference)** | | | | | | |
| Q2 | 0.08 (0.017) | <0.001* | -0.08 (0.018) | <0.001* | -0.05 (0.022) | 0.012* |
| Q3 | 0.12 (0.017) | <0.001* | -0.05 (0.019) | 0.006* | -0.09 (0.022) | <0.001* |
| Q4 | 0.26 (0.018) | <0.001* | -0.04 (0.019) | 0.059 | -0.13 (0.022) | <0.001* |
| **Internal Validation (UKB): Model 2** | | | | | | |
| **CosinorAge** | **Self-Rated Health** | | **Grip Strength** | | **EQ-5D** | |
| | Coefficients (SE) | p-value | Coefficients (SE) | p-value | Coefficients (SE) | p-value |
| CAA (Continuous) | 0.09 (0.006) | <0.001* | -0.03 (0.06) | <0.001* | -0.05 (0.007) | <0.001* |
| **CAA (Quartiles) (Q1: Reference)** | | | | | | |
| Q2 | 0.08 (0.017) | <0.001* | -0.08 (0.018) | <0.001* | -0.05 (0.021) | 0.015* |
| Q3 | 0.12 (0.017) | <0.001* | -0.04 (0.018) | 0.015* | -0.09 (0.021) | <0.001* |
| Q4 | 0.25 (0.017) | <0.001* | -0.03 (0.018) | 0.087 | -0.13 (0.022) | <0.001* |
| **Internal Validation (UKB): Model 3** | | | | | | |
| **CosinorAge** | **Self-Rated Health** | | **Grip Strength** | | **EQ-5D** | |
| | Coefficients (SE) | p-value | Coefficients (SE) | p-value | Coefficients (SE) | p-value |
| CAA (Continuous) | 0.09 (0.006) | <0.001* | -0.08 (0.007) | <0.001* | -0.05 (0.008) | <0.001* |
| **CAA (Quartiles) (Q1: Reference)** | | | | | | |
| Q2 | 0.08 (0.017) | <0.001* | -0.12 (0.017) | <0.001* | -0.05 (0.021) | 0.011* |

| | | | | | | |
|---|---|---|---|---|---|---|
| Q3 | 0.12 (0.017) | <0.001* | -0.12 (0.018) | <0.001* | -0.10 (0.022) | <0.001* |
| Q4 | 0.25 (0.018) | <0.001* | -0.14 (0.019) | <0.001* | -0.14 (0.023) | <0.001* |

| External Validation (NHANES): Model 2 | | | | | | |
|---|---|---|---|---|---|---|
| **CosinorAge** | **Self-Rated Health** | | **Grip Strength** | | **ADL** | |
| | Coefficients (SE) | p-value | Coefficients (SE) | p-value | Coefficients (SE) | p-value |
| CAA (Continuous) | 0.09 (0.020) | <0.001* | 0.02 (0.019) | 0.416 | 0.08 (0.019) | <0.001* |
| **CAA (Quartiles) (Q1: Reference)** | | | | | | |
| Q2 | 0.07 (0.069) | 0.336 | -0.02 (0.042) | 0.664 | 0.03 (0.051) | 0.551 |
| Q3 | 0.07 (0.057) | 0.226 | 0.07 (0.033) | 0.037* | 0.06 (0.048) | 0.212 |
| Q4 | 0.29 (0.066) | <0.001* | 0.02 (0.050) | 0.736 | 0.21 (0.056) | 0.001* |
| **External Validation (NHANES): Model 3** | | | | | | |
| **CosinorAge** | **Self-Rated Health** | | **Grip Strength** | | **ADL** | |
| | Coefficients (SE) | p-value | Coefficients (SE) | p-value | Coefficients (SE) | p-value |
| CAA (Continuous) | 0.10 (0.022) | <0.001* | 0.02 (0.022) | 0.480 | 0.09 (0.020) | <0.001* |
| **CAA (Quartiles) (Q1: Reference)** | | | | | | |
| Q2 | 0.07 (0.070) | 0.306 | -0.02 (0.044) | 0.681 | 0.03 (0.049) | 0.535 |
| Q3 | 0.09 (0.055) | 0.126 | 0.08 (0.035) | 0.050 | 0.07 (0.050) | 0.185 |
| Q4 | 0.32 (0.068) | <0.001* | 0.02 (0.056) | 0.787 | 0.22 (0.057) | 0.001* |

Generalized linear models were adjusted for age, ethnicity/race, Townsend deprivation index (UKB only), baseline assessment center (UKB only), education, employment, body mass index, smoking status, alcohol consumption, and comorbidities. We account for complex survey design in NHANES. *CAA* CosinorAgeAdvance, *SE* standard errors, *EQ-5D* European Quality of Life-5 Dimensions 5-levels*, ADL* Activities of Daily Living, *HRQoL* Health-related quality of life. *p values < 0.05. We standardized the CAAs and outcomes to have a mean value of 0 and a standard deviation of 1. **Model 1** excluded participants with shift work history (UKB only). **Model 2** additionally adjusted for sleep disorders. **Model 3** additionally adjusted for circadian rhythm stability and fragmentation.

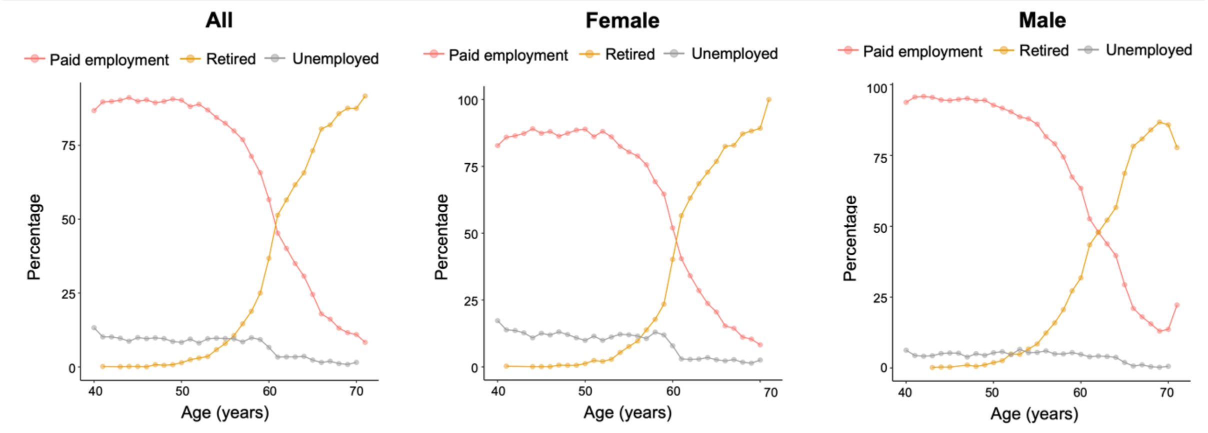


**Supplementary Fig. 3** Distribution of employment status in UK Biobank cohort.

**Supplementary Table 9.** International Classification of Diseases (ICD) codes used for identifying cause-specific mortality.

| Cause of Death | ICD-10 |
|---|---|
| CVD | I20-I25, I46, I50, I60-I64 |
| Cancer | C00-C97 |
| Diabetes | E10-E14 |
| Neurodegenerative | F01-F03, G122, G20, G21, G231-G233, G238, G239, G30, G31 |
| Respiratory | J40-J44, J47 |

**Supplementary Table 10.** Codes used for identifying prevalent and incident diseases in UK Biobank.

| Disease Type | Self-reported – touchscreen questionnaire | Self-reported – interview | Hospital records, Death records (ICD-10) | Cancer registry (ICD-10) |
|---|---|---|---|---|
| Case classification | Prevalent | Prevalent: diagnosis age < age, Incident: diagnosis age ≥ age | Prevalent: onset date of illness < start date of accelerometer use, Incident: onset date of illness ≥ start date of accelerometer use | |
| Hypertension | 6150 (4) | 20002 (1065,1072, 1073) | I10-I13, I15 | - |
| Diabetes | 2443 | 20002 (1220) | E10-E14 | - |
| CVD | 6150 (1,2,3) | 20002 (1066, 1074, 1075, 1076, 1081, 1086, 1491, 1583) | I20-I25, I46, I50, I60-I64 | - |
| Cancer | 2453 | 20001 | C00-C97 | C00-C97 |
| Neurodegenerative | - | 20002 (1259,1262,1263) | F01-F03, G122, G20, G21, G231-G233, G238, G239, G30, G31 | - |
| (i) Dementia/Alzheimer's/ Cognitive Impairment | | 20002 (1263) | F01-03, G30, G31 | - |
| (ii) Parkinson’s disease | | 20002 (1262) | G20, G21, G231-G233, G238, G239 | - |
| (iii) Motor neurone disease | | 20002 (1259) | G122 | - |
| Respiratory | 6152 (6) | 20002 (1112,1113,1472) | J40-J44, J47 | - |

**Supplementary Methods.** Derivation of CosinorAge

1. **Gompertz model**

$$CDF(x,t) = 1 - e^{\frac{-(e^{\gamma t}-1)(e^{bx})}{\gamma}}$$

$$f(x,t) = e^{\left[bx+rt-\frac{(e^{\gamma t}-1)(e^{bx})}{\gamma}\right]}$$

$$S(x,t) = e^{\frac{-(e^{\gamma t}-1)(e^{bx})}{\gamma}} = 1 - CDF(x,t)$$

*CDF(x,t)* is the cumulative distribution function. *f(x,t)* is the probability density function. *S(x,t)* is the survival function.
$bx = \sum_i^p x_i\, b_{i+}\, b_0$ where *p* is the number of predictors.
$\gamma$ = a shape parameter.

2. **Find parameters of Gompertz distribution**
We use maximum likelihood estimation (MLE) to estimate $b_{0,}b_{1,\ldots.}\ b_p \mid \gamma$.

$$argmax\ L(x_1, t_1, .., x_n^+, t_n^+) = \prod_{i=1}^{r} f(x_i, t_i) \prod_{i=r+1}^{n} S(x_i^+, t_i^+)$$

$^+$ indicates censoring.

To solve this, we can compute the derivative and solve for zero.

$$\frac{dL}{\beta_i} = 0, i = 0, \ldots, p$$
$$\frac{dL}{\gamma} = 0$$

3. **Compute mortality scores**
Since we now know $b_{0,}b_{p,}\ \gamma$, we compute the mortality score (MS) for a set of predictors $x_i$ for an individual *i* as follows:

MS = CDF(60, $x_i$ )

This represents the 5 year (60 months) mortality risk for an individual *i*.

Additionally, we fit a CDF(t, $CA_i$ ), where chronological age (CA) is the only predictor following steps 1-3 above. The goal is to find $b_{0,}b_{1,}\ \gamma$ for CA. This new parametric distribution we denoted from now on $CDF_{CA.}$

4. **Compute cosinor age**
Convert the mortality score into biological age (BA), so-called "CosinorAge", by executing

CDF(60, $x_i$ ) != $CDF_{CA}$(60, BA)

The obtained BA stands for "CosinorAge"
